# Numerical investigation of micro solid oxide fuel cell performance in combination with artificial intelligence approach

**DOI:** 10.1016/j.heliyon.2024.e40996

**Published:** 2024-12-06

**Authors:** Parastoo Taleghani, Majid Ghassemi, Mahmoud Chizari

**Affiliations:** a Department of Mechanical Engineering, K. N. Toosi University of Technology, Tehran, Iran; b School of Physics, Engineering and Computer Science, University of Hertfordshire, Hatfield, UK

**Keywords:** Micro solid oxide fuel cell, Proton-conducting electrolyte, Numerical model, Artificial intelligence, Artificial neural network

## Abstract

The current study presents a multiphysics numerical model for a micro-planar proton-conducting solid oxide fuel cell (H-SOFC). The numerical model considered an anode-supported H-SOFC with direct internal reforming (DIR) of methane. The model solves coupled nonlinear equations, including continuity, momentum, mass transfer, chemical and electrochemical reactions, and energy equations. Furthermore, The numerical model results are used in artificial intelligence (AI) models, the K-nearest neighbour (KNN) and, artificial neural network (ANN), to predict the current density and power density of the H-SOFC. The results show that increasing the air-to-fuel (A/F) ratio decreases the current density and overall cell power. In particular, improvements in power and current density observed in H-SOFC when the A/F ratio is set to 0.5, resulting in a respective increase of 2 % and 7 % compared to the initial state at A/F = 1. With an error rate of less than 1 % and an R-score of around 99 %, the ANN model shows good agreement with the numerical results.

## Nomenclature

**Variables**
V: Operating voltage
P: Power density
I: Current density
$E^{OCV}$: Open circuit voltage
R: Ideal Gas Constant
F: Faraday Constant
$p_{i}^{Ι}$: Species partial pressure at the electrode-electrolyte interface
I: Current Density
$l_{an}$: Anode Thickness
$l_{ele}$: Electrolyte Thickness
$l_{ca}$: Cathode Thickness
$D_{an,eff}$: Effective Anode Gas Diffusivity
$D_{ca,eff}$: Effective Cathode Gas Diffusivity
$j_{i}$: Mass-Flux Vector
$D_{i}^{e}$: Effective diffusion coefficient
$D_{i}^{m}$: Average diffusion coefficient
$D_{ij}$: Binary Diffusivity
$Q_{tot}$: Total heat generation
$k_{f}$: Gas mixture thermal conductivity
$k_{s}$: Solid thermal conductivity
$Q_{elec}$: Energy source of electrochemical reactions
$Q_{chem}$: Energy source of chemical reactions
$D_{i}^{kn}$: Knudsen diffusion
$r_{p}$: Average pore's radius
$k_{eff}$: Effective thermal conductivity of porous electrodes
U: Gas mixture velocity
$c_{p}$: Specific heat capacity

**Greek letters**
$\eta_{conc}$: concentration overpotential
$\eta_{ohm}$: Ohmic overpotential
$\eta_{act}$: Activation overpotential
$\sigma_{ele}$: Electrolyte Conductivity
$\phi_{e}^{an}$: Anodic Electrode Potential
$\phi_{e}^{ca}$: Cathodic Electrode Potential
$\sigma_{i}$: Ionic Conductivity
$\sigma_{e}$: Electrical Conductivity
ϕi: Ionic Potential
ϕe: Electrical Potential
$\alpha_{an}$: Anodic transfer coefficient
$\alpha_{ca}$: Cathodic transfer coefficient
$\omega_{i}$: Mass Fraction
$\mu_{i}$: Viscosity
ρ: Fluid Density
ε: Porosity
τ: Tortuosity
$\Omega_{D}$: Dimensionless diffusion collision

**Subscripts and superscripts**
ele: electrolyte
an: anode
ca: cathode
OCV: Open circuit voltage
Conc: concentration
act: activation

**Abbreviations**
AI: Artificial intelligence
KNN: K-nearest neighbours
ANN: Artificial neural network
DIR: Direct internal reforming

## 1 Introduction

Growing environmental concerns about fossil fuel resources have led to a significant focus on developing environmentally favourable power generation methods [[1], [2]]. Solid Oxide Fuel Cells (SOFCs) have gained significant attention due to their ability to efficiently convert chemical energy into electrical power through electrochemical reactions [3].

The SOFCs' high operational temperature range (600–1000 °C) provides several advantages in their applications [[4], [5]]. These benefits include a high rate of electrochemical reactions, high efficiency, adaptability to different fuels such as pure hydrogen [[6], [7]], biogas [8], natural gas, methanol, and ethanol [9], minimal pollutant emissions, the ability to function as a hybrid energy system [10], and various geometrical configurations [[11], [12], [13]]. However, the main obstacles to SOFC technology are the reduction of their cost and start-up time [14], improved durability [15], reduced degradation resulting from high temperatures, and enhanced SOFC system efficiency [5]. Direct internal reforming (DIR) has the potential to substantially decrease the overall cost and complexity of SOFC systems while simultaneously increasing their overall efficiency by utilizing the heat produced by the SOFC for endothermic reforming reactions in the anode [16]. Menon et al. [17] investigated H-SOFC systems with DIR numerically. The effect of different operational conditions on species transport, temperature distribution, and electrochemistry illustrated. The H-SOFC performance was analysed under various operating conditions, including the influence of partitioning the anode into multiple regions with various catalytic areas [17]. Kumuk et al. [18] created a computational model of an electrolyte-supported SOFC powered by hydrogen and coal gases with different electrolytes. The impact of temperature changes on the efficacy of proton-conducting and oxygen ion-conducting electrolyte SOFCs simulated numerically. It was demonstrated that O-SOFC was more efficient than H-SOFC at higher temperatures, while H-SOFC performed better at medium temperatures. Hydrocarbons and biomass are particularly suitable for SOFCs because of their low cost and wide availability [19]. However, the complex composition of these fuels results in multiple electrochemical and chemical reactions [20]. Gholaminezhad et al. [21] modified Fick's model to develop a 1D channel-level model of SOFC fuelled by methane. They simulated the electrochemistry and mass transfer phenomena of a SOFC to predict current density limitations. Min et al. [22] developed a 1D model for investigating the thermal and electrochemical characteristics of a SOFC stack. A parametric analysis performed to determine the optimal operating conditions of SOFCs by varying current density, fuel ratio, and pressure. The results indicated that high efficiency achieved using low current density, high fuel consumption, and low air usage. Tu et al. [23] looked into how the fuel composition, thermal efficiency, and electrical efficiency of SOFCs were affected by different ways of processing methane. They showed that steam reforming of methane produces more H2 and CO per mole of methane, resulting in high efficiency but low thermal efficiency. They showed that SOFCs can have high efficiency and low carbon deposition if the right O/C ratio chosen during the pretreatment of methane. This leads to the complexity of heat generation processes and complicates performance prediction and optimization [24]. Takino et al. [25] experimentally developed a modified equation for exchanging SOFC anode current density using methane fuel. The combination of their equation with numerical simulation used to investigate the efficiency factors of an electrolyte-supported SOFC. The modified equation reproduced the V-I characteristics and temperature distribution. Although computational fluid dynamics (CFD) has demonstrated high precision for evaluating performance, its complexity prevents online prediction and optimization. In contrast to the commonly used 2D or 3D multi-physics simulation (MPS) approach, by using artificial intelligence (AI) models, a black-box model is created by using a set of parameters for solving non-linear equation systems 10[[26], [27]]. Peksen et al. [28] investigated the effectiveness of the pre-reforming procedure for various syngas used as fuel by combining experimental data with numerical simulation methods. The thermochemistry of syngas fuel analysed using a CFD model. The developed model is then used to generate the necessary data to train a machine learning model. Additionally, studies looked at the combination of MPS and AI. As an example, a hybrid model for the investigation of SOFCs to address the challenge of long-term operation using difficult-to-use fuels was developed by Xu et al. [29]. The model combined MPS and deep learning, allowing for precise prediction with an error of less than 1 %. Also, they used a genetic algorithm to optimize, resulting in maximum power density while staying within the temperature gradient and operating condition limits. Song et al. [30] conducted experimental tests on 30 SOFC stack segments at varying furnace temperatures. Multiple evaluation criteria used along with ANN models to predict the stack's efficiency. The results indicated that the fitting errors of the three algorithms are within 5 %, whereas the neural network offered the best prediction accuracy in its results for generalizability and testing time. Yan et al. [31] presented a modelling framework to optimize the microstructures of SOFC electrodes using sequential simulations and multi-objective optimization assisted by artificial intelligence. They analysed the influence of various initial powder parameters, such as particle size distribution, on the SOFC's degradation rate and cathodic overpotential. They found that lower pore size and fine particle size result in a lower cathodic overpotential but a higher degradation rate. Xu et al. [32] developed a framework to enhance the performance of SOFCs using CFD modelling, ANN, and genetic algorithms. Initially, a 3D CFD model developed that considered multiple parameters, including geometry, microscopic features, and operating conditions, and data collected. Their results indicated that the ANN provided the most accurate predictions of SOFC performance, with an R-score value of 0.99889. Mahmood et al. [33] conducted a sensitivity analysis to explore the influence of key operational and design parameters such as operating temperature, material porosity, flow configurations, air-fuel ratios, and electrolyte thickness on the performance and thermal stresses within the SOFC's porous electrodes and solid electrolyte. Mütter et al. [34] optimized SOFC performance using ANN and genetic algorithms (GA). The ANN trained with data from a multi-physics model with molar fraction, temperature, and current density as the input data. The GA then applied to optimize power output, yielding near-global optimum solutions with alternative gas compositions. Gnatowski et al. [35] used an ANN model that dynamically updates the charge transfer coefficients based on operational conditions, trained on experimental data from SOFC anode polarization curves. The ANN predictions improved the accuracy of overpotential estimates, demonstrating its effectiveness in enhancing electrochemical modelling in SOFC applications [36]. Therefore, artificial intelligence provides a powerful prediction method for fuel cell applications. However, the performance of these applications depends on the appropriate choice of machine learning and deep learning technology [36]. AI technologies, specifically (ANNs), are being utilized to enhance the design and operational parameters of these fuel cells [37].

Due to the complexity of the governing equations in H-SOFCs, it demands a robust and efficient method for predicting performance under varying conditions. While traditional numerical simulations are accurate, they are often time-consuming and computationally intensive. This work aims to address this challenge by combining numerical modelling with AI techniques, K-nearest neighbours (KNN), and artificial neural networks (ANN) algorithms. This integration of AI offers an innovative approach to streamline the prediction of H-SOFC parameters like current density and power density, making it a valuable tool for rapid optimization and design in H-SOFC technology. This hybrid approach represents a step forward in leveraging AI to complement multiphysics simulations, providing more efficient and accurate performance predictions. From the review of the literature, it found that to date, little study conducted on the impact of A/F on the efficiency of proton-conducting solid oxide fuel cells (H-SOFCs). Therefore, the purpose of the current study is to conduct a comprehensive numerical investigation and analysis of how the A/F ratio affects H-SOFC performance. In the model, various parameters such as A/F, temperature, voltage, and fuel flow velocity considered for training the AI models. The model has been setup to solve the coupled non-linear governing equations, which include continuity, momentum, mass transfer, chemical and electrochemical reactions, and energy equations, by means of a multiphysics simulation method developed in house.

## 2 H-SOFC modelling

### 2.1 Model description

**Fig. 1:**
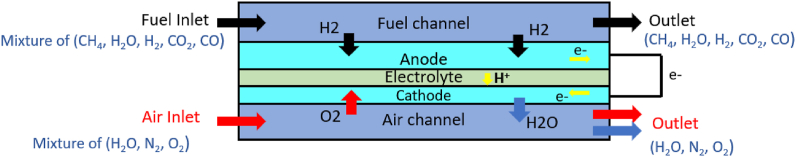
Representation of an anode-supported H-SOFC.

A multiphysics numerical simulation of a simplified micro-planar proton-conducting H-SOFC developed in the current study. The simplified H-SOFC model configured as shown in Fig. 1. It consists of a porous anode electrode, a porous cathode electrode, a solid electrolyte and channels for air and fuel. The geometric characteristics of the computational domain given in Table 1.

**Table 1:** Geometric characteristics of the present study.

Parameter	Values
Length of the cell	$2\times 10^{-2}$ (m)
Height of channels	$1\times 10^{-3}$ (m)
Anode height	$5\times 10^{-4}$ (m)
Electrolyte height	$1\times 10^{-4}$ (m)
Cathode height	$1\times 10^{-4}$ (m)

The numerical model solves the governing mathematical equations for the H-SOFC including continuity, momentum, mass transfer, chemical, and electrochemical reactions.

The H-SOFC functions through DIR process, where a mixture of hydrogen, methane, steam water, carbon dioxide, and carbon monoxide provided to the fuel channel. Hydrogen produced in the anode through chemical reactions, e.g. through the DIR process or water-gas shift reaction (WGSR). The DIR process can convert methane to a mixture of hydrogen and carbon monoxide (H2 and CO) on the surface of an anode, while the WGSR is a reversible chemical reaction that converts carbon monoxide and water to carbon dioxide and hydrogen. The chemical formulas for DIR and WGSR reactions are given in Eq. (1) and Eq. (2), respectively:

DIR:

(1) $$CH_{4}+H_{2}O\to 3H_{2}+\text{CO}$$

WGSR:

(2) $$\text{CO}+H_{2}O\to H_{2}+CO_{2}$$

The generated hydrogen is oxidized, as shown in Eq. (3):

(3) $$H_{2}↔2H^{+}+2e^{-}$$

Protons flow from the anode to the cathode through the proton-conducting electrolyte. At the cathode-electrolyte interface, the protons react with electrons received from the anode via an external circuit, as shown in Eq. (4):

(4) $$O_{2}+4H^{+}+4e^{-}↔2H_{2}O$$

The overall reaction of the SOFC is represented in Eq. (5):

(5) $${2H}_{2}+O_{2}↔2H_{2}O$$

### 2.2 The H-SOFC model assumptions

It has been assumed that the H-SOFC numerical model is operating in a steady state condition. The fluid flow is laminar and compressible, and all properties of the fluid change with temperature. The fluid behaves like an ideal gas. The electrolyte is considered dense and non-porous; therefore, there is no mass or momentum transfer through electrodes. Porous electrodes ohmic heating is not considered since the ionic conductivity is negligible compared to the electrical conductivity [38]. It assumed that electrodes have perfect selectivity for the electrochemical reactions, fuel undergoes electrochemical oxidation within the anode's porous electrode, and oxygen reduction occurs in the cathode's porous electrode.

### 2.3 Mathematical equations used in the model

The governing mathematical equations used to process the H-SOFC model are expressed as follows.

#### 2.3.1 Mass and momentum equations

The velocity field, *u*, and pressure, *P*, for the porous electrodes and gas channels are determined by applying continuity and momentum equations. The continuity equation is expressed in Eq. (6) [[39], [40]]:

(6) $$\nabla .\left(\rho u\right)=Q_{br}$$

Here ρ represents the mixture's density, and $Q_{br}$ represents the mass generated per unit volume. Since reactions only occur in electrode layers, $Q_{br}$ is equal to zero for gas channels. The momentum equations for the channels and electrodes are given in Eq. (7) and Eq. (8), respectively:

(7) $$\rho u\;.\nabla u=\nabla \left[\mu \left(\nabla u+{\left(\nabla u\right)}^{T}\right)-\frac{2}{3}\mu \left(\nabla .u\right)I\right]-\nabla p$$

(8) $$\frac{\rho }{\varepsilon }\left(u\;.\nabla \right)\frac{u}{\varepsilon }=\nabla .\left[\frac{\mu }{\varepsilon }\left(\nabla u+{\left(\nabla u\right)}^{T}\right)-\frac{2}{3}\frac{\mu }{\varepsilon }\;\left(\nabla .u\right)I\right]-\nabla p-\left(\frac{\mu }{\kappa }+\frac{Q_{br}}{\varepsilon^{2}}\right)u$$

Here *μ* is dynamic viscosity of a gas mixture, and *κ* and ε refer to the permeability and porosity of the electrodes, respectively [41]. The production and consumption of gas species that occur during chemical and electrochemical reactions lead to momentum sources at both electrodes [42].

#### 2.3.2 Electrochemical equations

The operating voltage at a specific current density is determined by Eq. (9):

(9) $$V=E^{OCV}-\left(\eta_{act}+\eta_{conc}\right)$$

In which $E^{OCV}$ is the cell's reversible open circuit voltage. The interface between the anode and air channel defined as ground; therefore, the anode open circuit voltage, $E_{an}^{OCV}$, is zero. The cathode open circuit voltage, $E_{ca}^{OCV}$, is obtained by applying Nernst's equation in Eq. (10) [43].

(10) $$E_{ca}^{OCV}=1.253-0.00024516T-\frac{RT}{2F}\ln\left(\frac{p_{H_{2}O\left(ca\right)}^{I}}{p_{H_{2}\left(an\right)}^{I}\;{p^{I}}_{O_{2}\left(ca\right)\;}^{0.5}}\right)$$

Electrode-electrolyte interface partial pressure, $P^{Ι}$, is computed by using the transport model [44]. Here *F* is the Faraday constant and $\eta_{act}$, $\eta_{conc}$ represent the activation and concentration overpotential, respectively. The activation overpotential is calculated using Eq. (11) [45]:

(11) $$\eta_{act}=\phi_{e}-\phi_{i}-E^{OCV}$$

Here $\phi_{e}$ is the electronic potential and $\phi_{i}$ is the ionic potential. The concentration overpotentials for the anode, $\eta_{conc,an}$, and cathode, $\eta_{conc,ca}$, are obtained from Eq. (12) and Eq. (13), respectively [46]:

(12) $$\eta_{conc,an}=\frac{RT}{2F}\ln\left(\frac{p_{H_{2}\left(an\right)}}{p_{H_{2}\left(an\right)}^{Ι}}\right)$$

(13) $$\eta_{conc,ca}=\frac{RT}{2F}\ln\left({\left(\frac{p_{O_{2}\left(ca\right)}}{p_{O_{2}\left(ca\right)}^{Ι}}\right)}^{0/5}\left(\frac{\;p_{H_{2}O\left(ca\right)}^{Ι}}{p_{H_{2}O\left(ca\right)}}\right)\right)$$

The potential distribution of electronic, $\sigma_{i}$, and ionic charges, $\sigma_{e}$, for the electrolyte, cathode, and anode are expressed in Eqs. (14), (15), (16) [47]:

(14) $$\nabla .\left(-\sigma_{i}^{el}\nabla \phi_{i}^{el}\right)=0$$

(15) $$\nabla .\left(-\sigma_{i}^{an}\nabla \phi_{i}^{an}\right)=\nabla .\left(-\sigma_{e}^{an}\nabla \phi_{e}^{an}\right)=+i_{v,an}$$

(16) $$\nabla .\left(-\sigma_{i}^{ca}\nabla \phi_{i}^{ca}\right)=\nabla .\left(-\sigma_{e}^{ca}\nabla \phi_{e}^{ca}\right)=-i_{v,ca}$$

The charge source term, $i_{v}$, is determined the Butler-Volmer equation, as expressed in Eq. (17) [45]:

(17) $$i_{v}=i_{0,electrode}\left[\exp\left(\frac{2\alpha_{an}F}{RT}\eta_{act}\right)-\exp\left(\frac{-2\alpha_{ca}F}{RT}\eta_{act}\right)\right]$$

Here $\alpha_{an}$ and $\alpha_{ca}$ are anode and cathode charge transfer coefficients.

#### 2.3.3 Mass transfer equations

The mass fraction species, $\omega_{i}$, in the electrodes and gas channels is determined by Eq. (18) [[36], [43]]:

(18) $$\frac{\partial }{\partial t}\left(\rho \omega_{i}\right)+\nabla .\left(\rho \omega_{i}u\right)=-\nabla .j_{i}+R_{i}$$

The diffusion mass-flux vector, $j_{i}$, is calculated using the modified Fick's equation, as represented in Eq. (19) [[44], [45]]:

(19) $$j_{i}=-\left(\rho D_{i}^{e}\nabla \omega_{i}+\rho \omega_{i}D_{i}^{e}\frac{\nabla M_{n}}{M_{n}}-j_{c,i}+D_{i}^{T}\frac{\nabla T}{T}\right)$$

The species mass source term, $R_{i}$, is calculated according to the values of DIR rate, $R_{DIR}$, and WGSR rate, $R_{WGSR}$, in electrodes [48]. The values of $R_{i}$ for chemical and electrochemical reactions are obtained from Eq. (20) and Eq. (21), respectively [[37], [47]]*:*

(20) $$R_{i}=\omega_{i}M_{i}\left(a_{i}R_{DIR}+b_{i}R_{WGSR}\right)$$

(21) $$R_{i}=\omega_{i}M_{i}\frac{c_{i}i_{v}}{nF}$$

As a result, the overall mass generation term is computed using Eq. (22):

(22) $$Q_{br}=\sum_{i}R_{i}$$

In porous electrodes, Knudsen diffusion, $D_{i}^{Kn}\text{,}$ should add to the average diffusion coefficient, $D_{i}^{m}\text{,}$ due to considerable species collisions with the walls. Therefore, the effective diffusion coefficient, $D_{i}^{e}$, is calculated using the Bosanquet formula, as shown in Eq. (23) [49]:

(23) $$\frac{1}{D_{i}^{e}}=\frac{1}{D_{i}^{m}}+\frac{1}{D_{i}^{Kn}}$$

Where $D_{i}^{Kn}$ and $D_{i}^{m}$ are calculated using Eq. (24) and Eq. (25), respectively [[50], [51]]:

(24) $$D_{i}^{Kn}=\frac{2}{3}\frac{\varepsilon }{\tau }\;r_{p}\sqrt{\frac{8RT\;}{\pi W_{k}}}$$

(25) $$D_{i}^{m}=\frac{1-\omega_{i}}{\sum_{i\neq j}^{K_{g}}\omega_{j}/\gamma D_{ij}}$$

The binary diffusion coefficient, $D_{ij}$, is determined by the Maxwell-Stefan equation, and *γ* equals one [43]. Here, *τ* is the tortuosity of porous electrodes, and $r_{p}$ stands for the average pore's radius.

#### 2.3.4 Energy equations

The temperature profile across the entire domain is determined as shown in Eq. (26) [52]:

(26) $$\rho c_{p}u\;.\nabla T+\nabla .\left(-k_{eff}\nabla T\right)=Q_{tot}$$

Here, $c_{p}$ is the specific heat, and $k_{eff}$ is the thermal conductivity coefficient [53]. The mass source term of the energy equation, $Q_{tot}$, is given as follows in Eqs. (27), (28), (29) [[48], [54]]:

In electrolyte:

(27) $$Q_{tot}=\sigma_{i}^{el}{\left(\nabla \phi_{e}^{el}\right)}^{2}+Q_{elec}$$

In Cathode*:*

(28) $$Q_{tot}=\sigma_{i}^{ca}{\left(\nabla \phi_{e}^{ca}\right)}^{2}+\sigma_{e}^{ca}{\left(\nabla \phi_{e}^{ca}\right)}^{2}+i\eta$$

In anode*:*

(29) $$Q_{tot}=\sigma_{i}^{an}{\left(\nabla \phi_{e}^{an}\right)}^{2}+\sigma_{e}^{an}{\left(\nabla \phi_{e}^{an}\right)}^{2}+i\eta +Q_{chem}$$

Where *i* represents the electrode current density and *iη* is the heat generated from irreversible overpotential losses. Here, *σ(∇φ)*^*2*^ illustrates the Ohmic heating term, and $Q_{chem}$ is the energy source term related to chemical reactions. $Q_{elec}$ and $Q_{chem}$ are energy sources for electrochemical and chemical reactions, respectively [[37], [46]].

### 2.4 Boundary conditions of the H-SOFC model

Table 2 presents the operational conditions and material properties used in the current study [[17], [54]]. To solve the governing equations, the following boundary conditions considered: At the inlet of gas channels, the velocity field, temperature, and gas mixture composition specified. At the outlet, atmospheric pressure and zero mass diffusion assumed. The fluid regime is continuous, and the outer walls have no-slip boundary conditions and are thermal insulation.

**Table 2:** The operational conditions and material properties used in current study [[17], [54]].

Parameter	value
Operational conditions	
T	973 K
$P_{\text{in}}$	1 atm
$P_{\text{out}}$	1 atm
$V_{\text{Fuel}}$	1–3 m/s
$V_{\text{air}}$	3 m/s
Mole fraction of input fuel components	$H_{2}$ 0.661 ، $CH_{4}$ 0.116، $H_{2}O$0.003، CO 0.218, CO_2_ 0.002
Mole fraction of input air components	0.001H_2_O ،0.789 N_2_ ، 0.21 O_2_
**Material properties**	
porosity of electrodes	0.4
Permeability	$10^{-12}$
Electrode's tortuosity	3
Thermal conductivity of the electrolyte	2.16W/m.K
Anode thermal conductivity	1.86W/m.K
Cathode thermal conductivity	5.84W/m.K
Current density of anode exchange	$5300\;A/m^{2}$
Current density of cathode exchange	$2000\;A/m^{2}$
Electrolyte conductivity	0.009T−6.157S/m
Density of SOFC components	$452.63\;\text{kg}/m^{3}$
Specific heat capacity of SOFC components	3515.75J/kgK
Pore radius	0.5μm
$D_{\text{an},\text{eff}}$	$8.984\times 10^{-5}m^{2}/s$
$D_{\text{Ca},\text{eff}}$	$4.748\times 10^{-6}m^{2}/s$
$\sigma_{\text{ele}}$	$225.92\;\exp\left(-6.3\times 10^{3}/T\right)\Omega^{-1}m^{-1}$

## 3 Numerical procedure

**Fig. 2:**
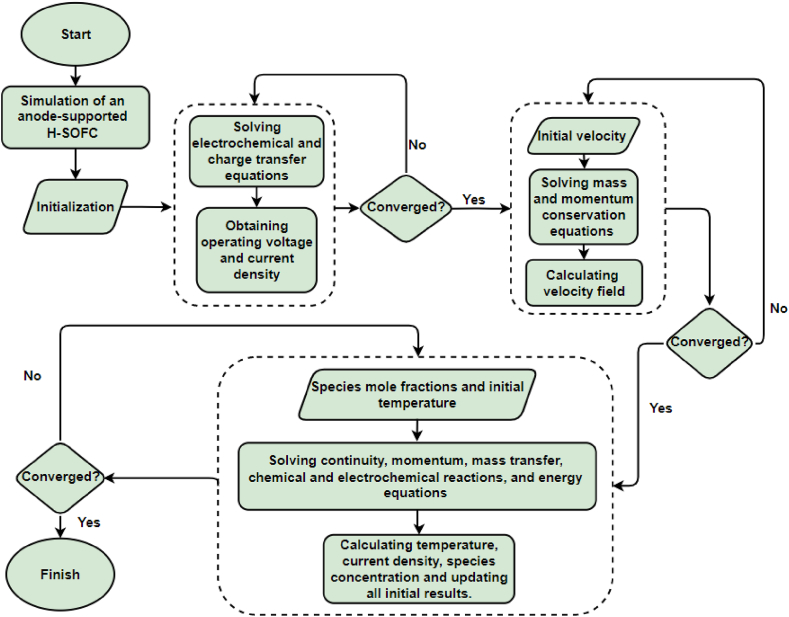
Diagram of the H-SOFC modelling process.

The H-SOFC model uses discretised geometry to apply the previously introduced nonlinear equations to discretised nodes and mesh elements. Initially, input parameters defined within the numerical model to develop the electrochemical equations and obtain initial solutions for the operating voltage and cell's current density. In the next step, the mass and momentum conservation equations solved to obtain the velocity field. In final stage, all models used to solve the coupled partial differential equations simultaneously. The model then updates initial solutions and calculates all outputs. This approach involves solving the independent nonlinear partial differential equations individually and using their results as initial values for all the governing equations. Iterations repeated in each step until convergence achieved. Fig. 2 provides an overview of the overall process of the H-SOFC modelling process, including all the essential steps.

## 4 Model validation

**Fig. 3:**
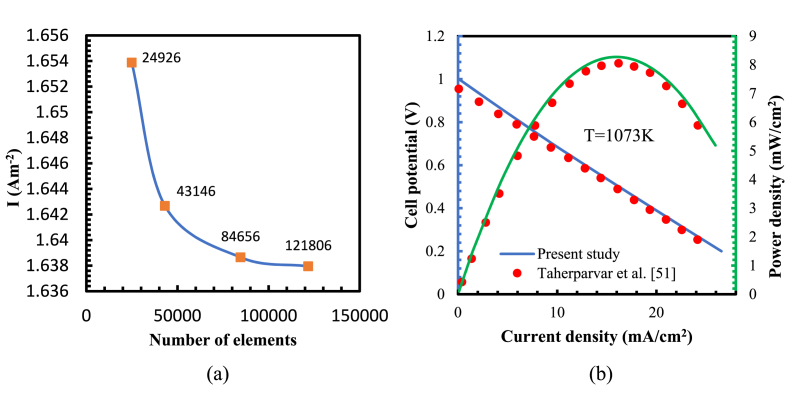
(a) Comparison of average current density along the electrodes with different grid sizes, (b) Comparison of multiphysics simulation polarization curves and experimental data.

A grid independence test conducted to determine the influence of the mesh size on the output current density and select the optimal grid size for the present study. Four different computational grids with different element sizes analysed, as shown in Fig. 3(a). The results reveal no notable difference (less than 3 %) in the current density values between a computational mesh of 84,656 elements and 121,806 elements. Consequently, a mesh size of 84,656 elements chosen for all simulations. To validate the numerical simulation, a comparison is made between the polarization curves of the numerical results and the results obtained from an experiment conducted by Taherparvar et al. [55], as depicted in Fig. 3(b). The geometric parameters, operating conditions, and cell materials kept consistent.

## 5 Artificial intelligence (AI)

Due to the non-linear and complex nature of the governing equations within the H-SOFC numerical model, running the model for different conditions would be costly. However, a trained AI tool may be able to analyse the performance of the model under different considerations. This study contains a combination of multiphysics simulation and AI techniques. Initially, the data obtained from numerical simulations utilized to train and the AI model. An artificial neural network (ANN) k-nearest neighbours (KNN) algorithm, which involves preprocessing the data, splitting it into training and testing sets, and normalizing it. We ran 364 simulation with different values of some H_SOFC parameters(temperature, air-to- fuel ratio, velocity of the fuel gas, and voltage.

### 5.1 Data preprocessing

Before constructing the AI models, the input parameters obtained from the results of H-SOFC numerical simulations, including the air-to-fuel ratio, voltage, temperature, and input fuel velocity, normalised within a range of zero to one. The outputs considered in this study are the H-SOFC current density and power density. For network training purposes, 364 data sets used, which randomly split into two groups: a training set (composed of 70 % of the data) and a testing set (consisting of 30 % of the data). The input parameters and their range of values shown in Table 3. It is worth noting that the data analyses in this study performed using Python, which is an open-source high-level programming language widely used in scientific computing. The machine learning models implemented using the Keras and Scikit-learn libraries written in Python.

**Table 3:** Variations in the input parameters for KNN and ANN models.

Input parameters	Value
Air-to-fuel ratio	0.5–4
Voltage	0.1–1.1(v)
Temperature	800-973(K)
Inlet fuel velocity	1-3(m/s)

### 5.2 KNN model

The K-nearest neighbour algorithm is a machine learning method used to classify new data points by comparing them to the nearest data points in the training dataset. The K-nearest neighbour algorithm enables the consideration of K arbitrary neighbours. The value of K represents the number of neighbours that considered. To determine the class of each data point, the algorithm considers the neighbouring data points of its surrounding class. The predicted class assigned based on the class with the highest count among the neighbours. In this study, the value of K is determined based on the minimum error obtained for each K value.

### 5.3 ANN model

The artificial neural network is a supervised learning method consisting of interconnected neurons with adjustable weights that process data through three or more layers. The components of an ANN include an input layer, one or more hidden layers, an output layer, a set of neurons, weights, biases, and activation functions [56]. A structure of ANN with two hidden layers shown in Fig. 5. The model selection procedure is the most crucial aspect of a neural network, as it directly influences the model's output. Various architectural and hyperparameter configurations must be explored and optimized to determine the optimal model, such as the number of input parameters, number of neurons, number of hidden layers, activation functions, and loss functions [57].

#### 5.3.1 Hyperparameter tuning

For the ANN model, various architectures and hyperparameters (such as the number of hidden layers, number of neurons, activation functions, etc.) need to be optimized to ensure high accuracy. We used the grid search method to find the optimal values for our model. Table 4 shows the different values of hyperparameters that evaluated to find the ultimate values.

**Table 4:** Hyperparameter tuning with grid search.

Hyper parameters	Tested values	Optimal value
Learning rate	0.1, 0.01, 0.003	0.01
Number of hidden layers	1, 2, 3	2
Number of neurons	16, 32, 64, 128	(32,64)
Batch size	4,16, 32, 64	16
Epochs	100, 200, 300	200
Activation function	Relu, Sigmoid, Softmax	Relu

**Fig. 4:**
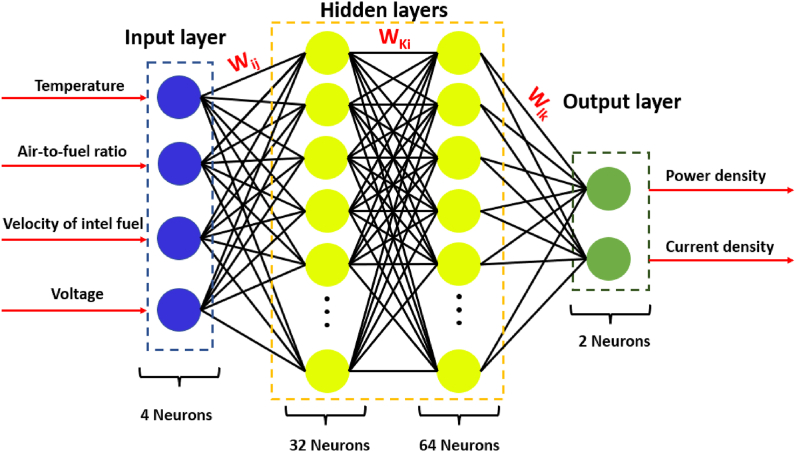
A structure of ANN with two hidden layers for the current study.

Fig. (4) illustrates the structure of the ANN used in this study, including an input layer, two hidden layers, and an output layer, along with the number of neurons in each layer. Additionally, the input and output data are depicted in Fig. 4. The final hyperparameter values of the optimized ANN model are presented in Table 5.

**Fig. 5:**
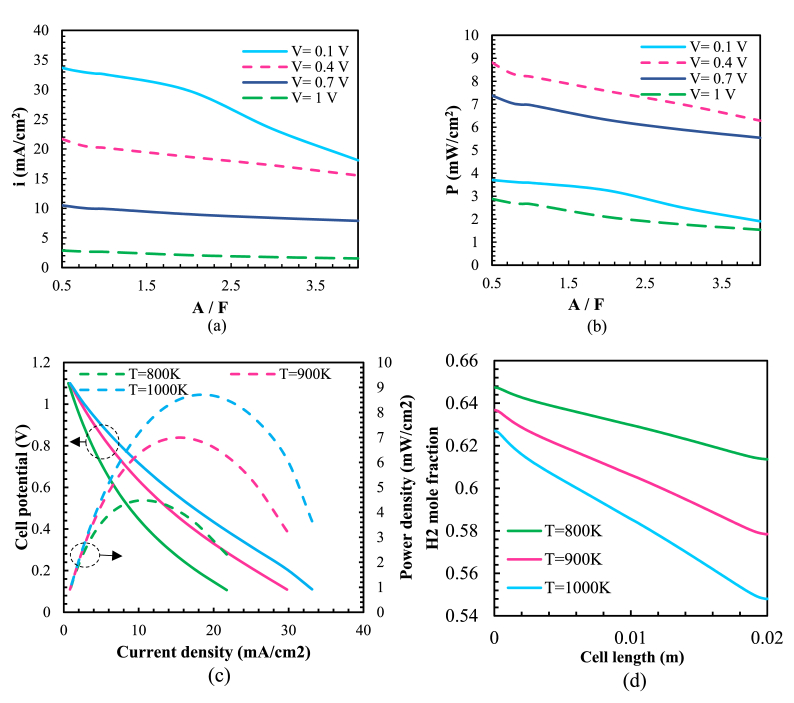
(a) The current density distribution at various A/F ratios; (b) distribution of power density at various A/F ratios at a temperature of 973 K*; (c)* V-I and P-I curves with a fuel-to-air ratio of one at different temperatures; (*d*) H2 mole fraction variations at the anode-electrolyte interface for various temperatures.

**Table 5:** Hyperparameters for training ANN models.

Output	Model	Input parameters	Number of neurons in hidden layers	Output activation function	Batch size
Prediction of power density	ANN) first model(	T, v(m/s), S/V, V(v)	(32, 64)	Sigmoid	16
	ANN)second model(	T, v(m/s), S/V, V(v)	(32, 64, 32)	Sigmoid	32
Prediction of current density	ANN)second model(	T, v(m/s), S/V, V(v)	(32, 64)	Sigmoid	32

### 5.4 Evaluation of AI models

To evaluate the accuracy of trained models, some standard criteria are used, which are [58]:

Mean Absolute Error (MAE): The mean absolute value of the prediction errors, regardless of their direction. The smaller (closer to 0) the value, the better the trained model performs. The MAE is expressed as in Eq. (30):

(30) $$MAE=\frac{\sum_{i=1}^{n}\left|y_{i}-x_{i}\right|}{n}$$

Where x is the predicted value and y is the actual value.

Mean Squared Error (MSE): This error is similar to the MAE, but it squares the absolute values of the errors. However, it is typically more challenging to interpret due to the magnitude of the values and their dissimilarity to the data. The MSE value is calculated using Eq. (31):

(31) $$MSE=\frac{\sum_{i=1}^{n}{\left(x_{i}-y_{i}\right)}^{2}}{n}$$

Root Mean Squared Error (RMSE): This type of error addresses the interpretation problem of MSE by taking the square root of the final value, so that the resulting error is of the same data type as the original data. The RMSE value is calculated using Eq. (32):

(32) $$RMSE=\sqrt{\sum_{i=1}^{n}\frac{{\left(x_{i}-y_{i}\right)}^{2}}{n}}$$

R-squared ($R^{2}$): This measure demonstrates the correlation between the model outputs and the predicted values. It is important when a statistical model is used for prediction or for evaluating test data. The closer the value is to one, the higher the model's accuracy. The R-squared value is calculated using Eq. (33):

(33) $$R^{2}=1-\sum_{i=1}^{n}{\left(x_{i}-y_{i}\right)}^{2}/\sum_{i=1}^{n}{\left(x_{i}-\bar{x_{i}}\right)}^{2}$$

## 6 Results and discussion

Different operating conditions are the main factors affecting the electrochemical performance of SOFCs. In this part of the study, the effects of various operating parameters (e.g., operating temperature, air/fuel ratio), the Effects of variation in inlet fuel velocity, and the prediction of fuel cell current and power density by an ANN model are investigated. The study's results categorized into two main groups: numerical simulation results and AI results.

### 6.1 Numerical simulation

Multiphysics simulation results presented as follows.

#### 6.1.1 A/F ratio effects

The effect of different air-to-fuel (A/F) ratios on cell performance studied by simulating the model at a temperature of 973 K with varying ratios of A/F ranging from 0.5 to 4. This ratio obtained by changing the value of the fuel. Fig. 5(a) displays the current density versus the air-to-fuel ratio for different voltages: 0.1 V, 0.4 V, 0.7 V, and 1 V. The fuel cell's current density decreases with an increasing A/F ratio. Variations in current density reduction are more significant at lower voltages, particularly at V = 0.1 and a higher A/F ratio. The highest current density of 33.6 mA/cm2 achieved at A/F = 0.5 and V = 0.1. This decrease in current density attributed to fuel reduction as it moves along the fuel channel, leading to a decline in reaction and current density. Fig. 5(b) shows the power density versus the air-to-fuel ratio for various voltages, including 0.1 V, 0.4 V, 0.7 V, and 1 V. The fuel cell's power density decreases as the A/F ratio increases. For instance, at 0.4 V, by increasing the A/F ratio from A/F = 1 to A/F = 4, the cell's maximum power reduced by about 20 %. At higher A/F ratios, the decrease in power density becomes more significant as the fuel entering the fuel channel leads to fuel dilution, affecting both reforming and electrochemical reactions. Consequently, a higher A/F ratio decreases the rate of both reactions.

Fig. 5(c) shows the variations in voltage-current and power-current density for an A/F ratio 1 at different temperatures. Increasing the temperature has a significant impact on the output power and current density, resulting in an overall increase in cell efficiency. The findings show that when the temperature decreases from 1000K to 800K, the output power and current density decrease by 48 % and 41 %, respectively. In Fig. 5(d), the hydrogen mole fraction variation at the anode-electrolyte interface shown as a function of temperature. Where the temperature rises, there would be an increase in the variation of the H2 mole fraction. For example, variation in the H2 mole fraction at 1000K is approximately 3.5 percent higher than T = 800K. As temperatures rise, the rate of electrochemical processes increases, leading to greater fuel consumption. Furthermore, the variation in H2 mole fractions along the cell length at T = 1000K is 7 percent greater than the corresponding value at T = 800K. To confirm the accuracy of the numerical modeling, simulation results are compared with literature papers. Findings align with [[50], [59]], which highlight that higher operating temperatures enhance current density, and power density, and reduce ohmic losses. This is also corroborated by Refs. [[33], [60]] who showed that air-fuel ratios impact overall SOFC performance, although its effect is smaller than temperature.

#### 6.1.2 Effects of variation in inlet fuel velocity

**Fig. 6:**
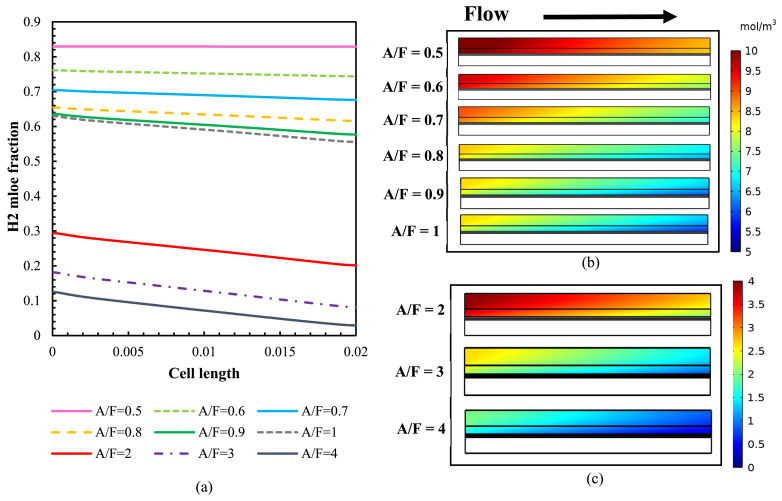
(a) H_2_ mole fraction variation at the anode-electrolyte interface with varying A/F ratio at a temperature of 973K, (b); (c) H_2_ mole fraction distribution in the anode and fuel flow channel at a temperature of 973K as a function of different A/F ratios.

Fig. 6(a) shows the hydrogen mole fraction distribution at the anode-electrolyte interface during operation at a voltage of 0.5 V with an inlet fuel velocity of 1. The results demonstrate that a higher A/F ratio leads to more significant variations in the H2 mole fraction. An A/F ratio of 4 has the most variation in H2 concentration, from a maximum of 0.125 at the inlet to a minimum of 0.029 at the outlet. In Fig. 6(b) and (c), the distribution profiles of hydrogen concentration in the anode and fuel flow channels presented for different A/F ratios at a temperature of 973K and a voltage of 0.5 V. As the A/F ratio doubles, triples, and quadruples, the hydrogen concentration drops at the anode outlet, reaching 36 %, 15 %, and 5.5 % of the hydrogen concentration at the anode outlet with an A/F ratio of 1, as shown in Fig. 6(c). For an A/F ratio of 0.5, the maximum H2 mole fraction is 0.8291 at the outlet and decreases to 0.029 for an A/F ratio of 4.

### 6.2 AI results

As mentioned, fuel cell power and current density predictions made using ANN and KNN methods. The results obtained from these methods presented below.

#### 6.2.1 Prediction of power

**Fig. 7:**
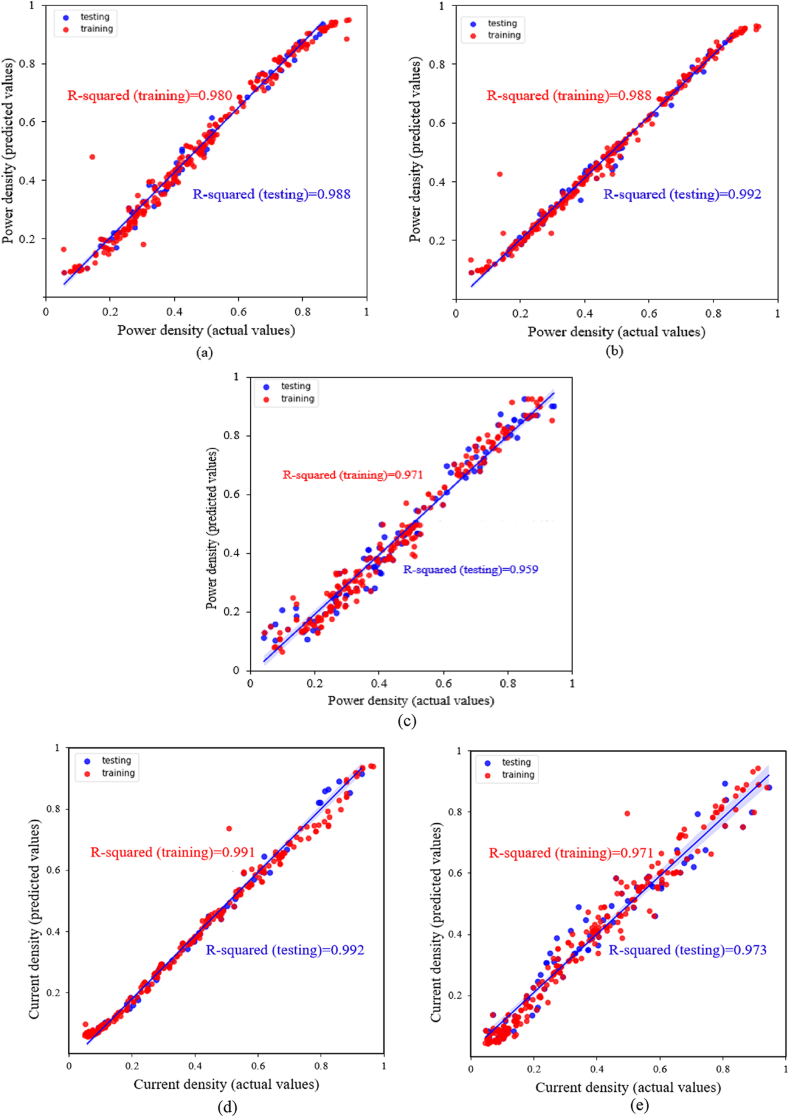
(a) ANN model with three hidden layers and the output parameter P; (b) ANN model with two hidden layers and the output parameter P; (c) The KNN model with K = 3 and the output parameter P; (d) ANN model with two hidden layers and the output parameter I; (e) KNN model with K = 3 and the output parameter I.

For power prediction, 364 data points used. Fig. 7 compares the expected power with the actual power determined by numerical results for both the training and test set. In part (a), the ANN model with three hidden layers can achieve an MAE of 0.031200 and a $R^{2}$ coefficient of 0.98 for test data. As shown in Fig. 7(b), the ANN model with two hidden layers achieves an MAE of 0.01612 and a $R^{2}$ coefficient of 0.99 for test data, indicating improved accuracy compared to the first model (the ANN with three hidden layers).

The results of using the KNN model to predict the cell's power density shown in Fig. 7(c). The optimal value for K, which determines the number of nearest neighbours considered, is determined by calculating the MAE for K values ranging from 1 to 20. Among the range of K values evaluated, K = 3 obtains the lowest MAE of 0.036127, and thus, it selected for training the present model. The accuracy obtained for the training and testing datasets is 97 % and 95 %, respectively. However, the KNN model exhibits lower accuracy compared to the ANN model.

Choosing the optimal model to predict a target quantity is a procedure that requires attention. In Table 6, the performance of various trained ANNs and KNN models for predicting the power density examined following hyperparameter tuning. According to the results, the ANN model with two hidden layers achieves the best accuracy.

**Table 6:** Evaluation of the trained models' performance for predicting power density.

method	MAE	MSE	RMSE	$R^{2}$
ANN (First model)	0.016129	0.0006539	0.0255730	0.990
ANN (Second model)	0.031200	0.0017748	0.042128	0.980
KNN	0.036127	0.0032140	0.056692	0.950

#### 6.2.2 Prediction of fuel cell current density

Fig. 7(d) and (e) compare the current density values predicted by the ANN and KNN models with the actual values (obtained from the numerical data). The outcomes demonstrate similar result as power prediction, the ANN has the best accuracy, and the distribution of training and testing data points is more uniform around the y = x line.

Table 7 presents the MAE, MSE, RMSE, and R^2^ coefficients for both the ANN and KNN models.

**Table 7:** Evaluation of trained models for predicting current density.

Method	MAE	MSE	RMSE	$R^{2}$
ANN	0.016599	0.0005871	0.024231	0.99
KNN	0.026136	0.0015718	0.039646	0.97

The results show that the ANN model is more accurate and has fewer errors than the KNN model.

Three new data points have used to evaluate the accuracy of the selected, trained model (ANN with two hidden layers) in predicting target values. The input values for these three test data sets presented in Table 8.

**Table 8:** Input parameter values for three new data points.

The values of input parameters				
Test data number	(°C)Temperature	(m/s)Velocity of inlet fuel	Air-to-fuel ratio	(V)Voltage
Data point 1	850	2.5	1.5	0.1–1.1
Data point 2	950	2.5	1.75	0.1–1.1
Data point 3	910	2	2	0.1–1.1

**Fig. 8:**
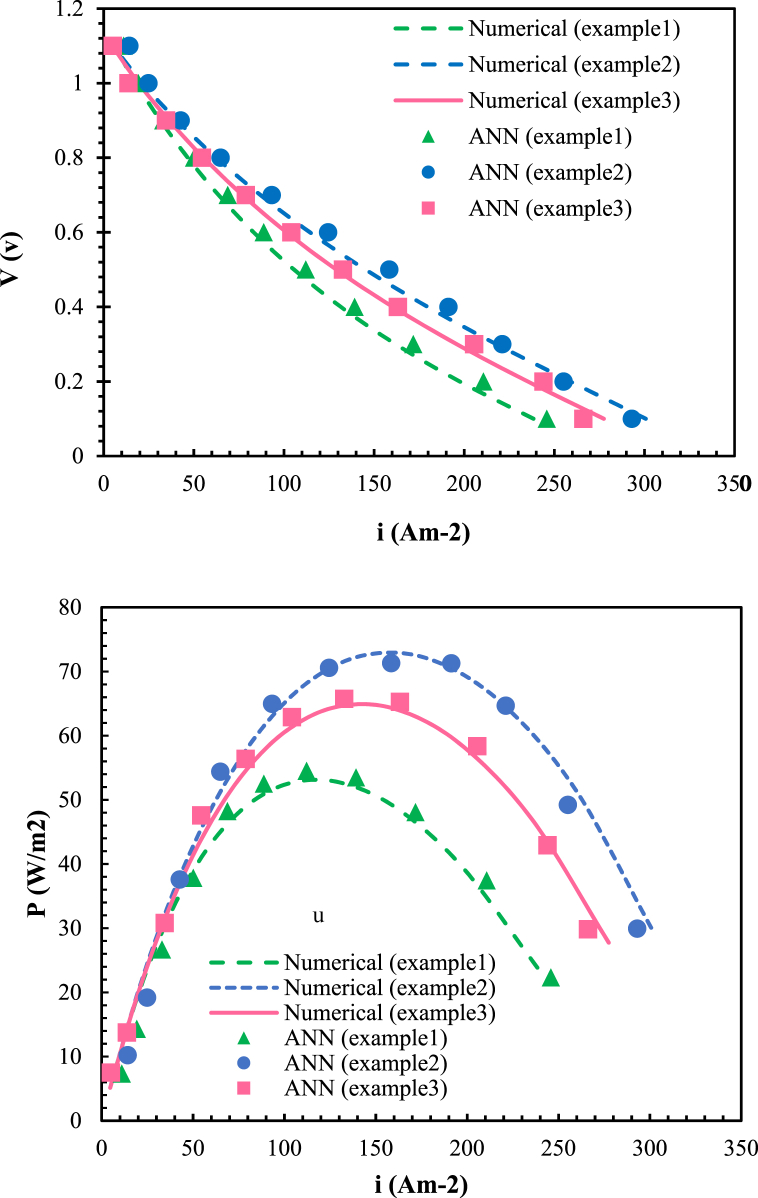
The predicted value by the ANN model and its comparison with the actual value obtained from the simulation results (a) of current density and (b) of power density.

Fig. 8 provides a comparison between the results obtained from the multi-physics model simulation and the predicted outcomes from the first ANN model, which demonstrates higher accuracy. This trained ANN model effectively predicts variations in current density (Fig. 8(a)) and power density (Fig. 8(b)) values based on voltage changes. In comparison to other research, the ANN method used in this paper demonstrated excellent predictive accuracy for current and power density with an error rate below 1 % and an R-score of approximately 99 %. Similar results were found by Xu et al. [29] and Wang et al. [3]who combined multi-physics simulations with deep learning, achieving a prediction error of less than 1 %. These studies confirm the reliability of AI models in SOFC prediction and highlight the potential for further optimization through advanced algorithms.

## 7 Conclusion

This paper presents a comprehensive investigation combining numerical analysis and (AI) techniques to study and predict the performance of a micro proton-conducting solid oxide fuel cell (H-SOFC) fuelled with methane. Using a detailed numerical approach, we solved complex mathematical equations, including electrochemical, mass transfer, heat transfer, continuity, and momentum equations, to understand the behaviour of H-SOFCs under varying operational conditions and by changing the values of air to fuel ration(A/F), temperature, velocity of the fuel gas and voltage. The numerical simulation results used to train both an artificial neural network (ANN) and a K-nearest neighbours (KNN) model, enabling accurate predictions of the cell's output power and current density.

The main findings of this study summarised as follows.

• Impact of Temperature: The performance of H-SOFC using DIR of methane fuel improves significantly as temperature increases. The simulation results show that as the operating temperature increases from 800 K to 900 K and 1000 K, the maximum output power density increases from 74.4 mW/cm^2^ to 678.8 mW/cm^2^ and 932.6 mW/cm^2^, respectively, indicating a substantial enhancement in cell performance.
• Effect of Air-to-Fuel (A/F) Ratio: The numerical model reveals that the current density and power density of H-SOFC decrease as the A/F ratio increases. Optimal performance achieved at an aspect ratio of A/F = 0.5, where power density increases by 2 % and current density by 7 % compared to the state at A/F = 1. Conversely, at A/F = 4, power and current density decrease by approximately 25 % compared to A/F = 1.
• AI Model Accuracy: The ANN model demonstrated remarkable accuracy in predicting the power density and current density of the H-SOFC, with average absolute errors of less than 1.6 % and an R-score of about 99 %. This confirms the ANN model's potential as an effective tool for performance prediction, reducing the reliance on time-consuming numerical simulations.

Overall, increasing the temperature and decreasing the electrochemical conversion voltage enhances the hydrogen conversion rate, leading to a faster reaction of methane to hydrogen land, and resulting in improved fuel cell performance. The combination of numerical modelling and AI-based prediction represents a significant advancement in studying of H-SOFCs. This hybrid approach provides a deeper understanding of H-SOFC operations and offers an efficient and accurate method for predicting performance parameters, significantly reducing the computational cost. The results of this work have the potential to influence future research, promoting the development of more efficient, AI-assisted fuel cell technologies that are practical for a wide range of applications. Future research could explore the integration of H-SOFCs into hybrid energy systems, where the fuel cell works in conjunction with other energy technologies (such as gas turbines or renewable energy sources).

## CRediT authorship contribution statement

**Parastoo Taleghani:** Writing – original draft, Validation, Conceptualization, Writing – review & editing. **Majid Ghassemi:** Writing – review & editing, Supervision, Conceptualization. **Mahmoud Chizari:** Writing – review & editing, Supervision, Conceptualization.

## Ethics approval and consent to participate

Not applicable.

## Consent for publication

Not applicable.

## Availability of data and materials

Data is available upon request from the corresponding author.

## Funding

This research did not receive any specific grant from funding agencies in the public, commercial, or not-for-profit sectors.

## Declaration of competing interest

The authors declare that they have no known competing financial interests or personal relationships that could have appeared to influence the work reported in this paper.
