# Supplementary material for: Numerical investigation of micro solid oxide fuel cell performance in combination with artificial intelligence approach
Source: Heliyon. 2024 Dec 6;10(24):e40996. doi: 10.1016/j.heliyon.2024.e40996 (PMC11696671; doi:10.1016/j.heliyon.2024.e40996)
Supplement: Multimedia component 1 [file mmc1.docx]

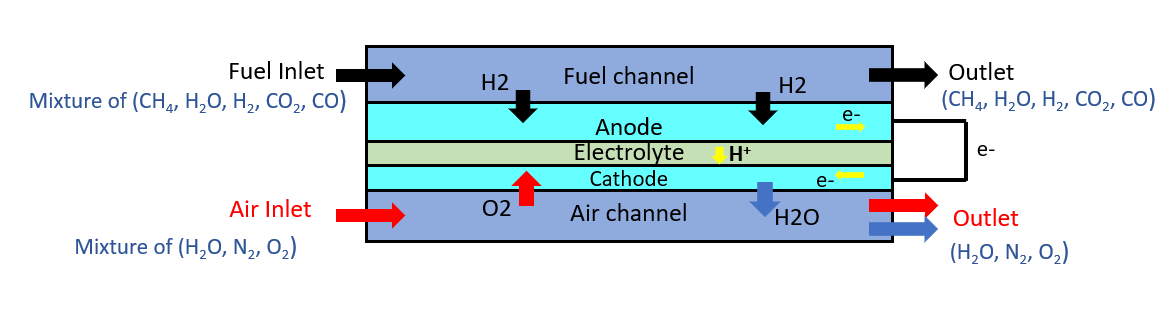


Fig. 1. Representation of an anode-supported H-SOFC.


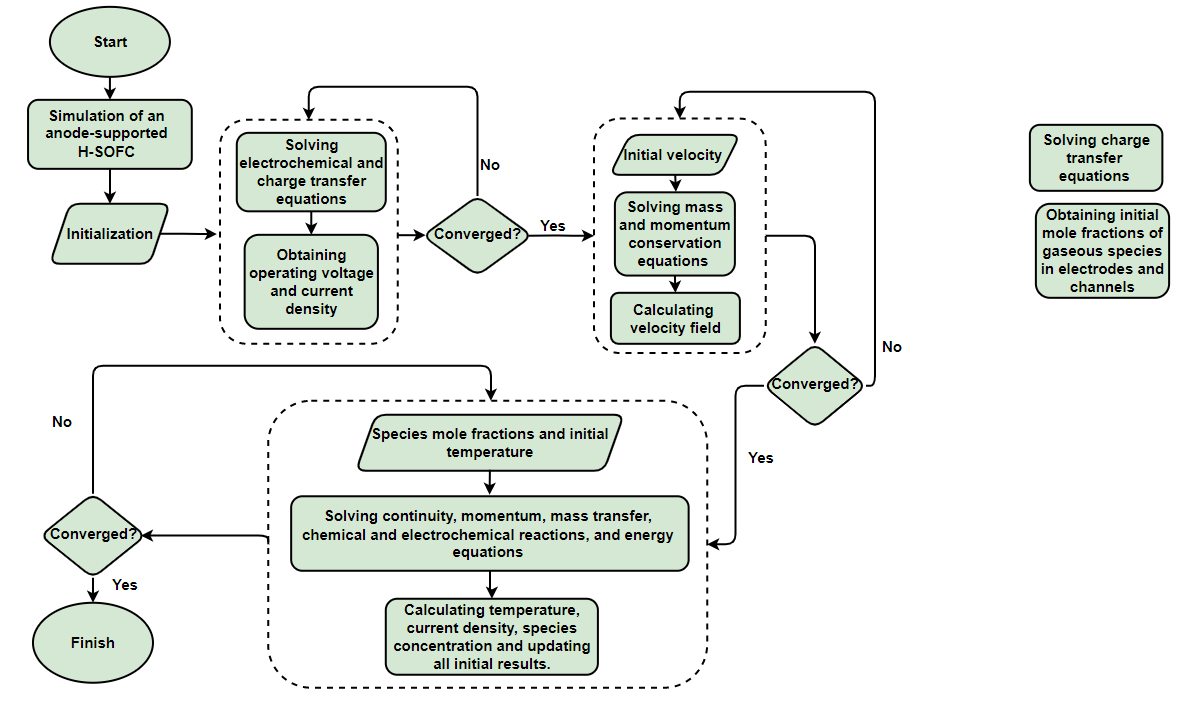


Fig. 2. Diagram of the H-SOFC modelling process

(a)


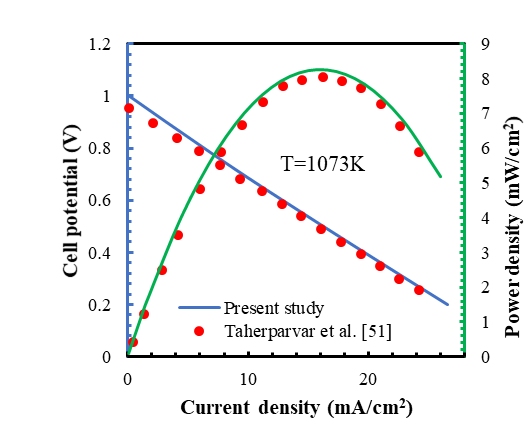

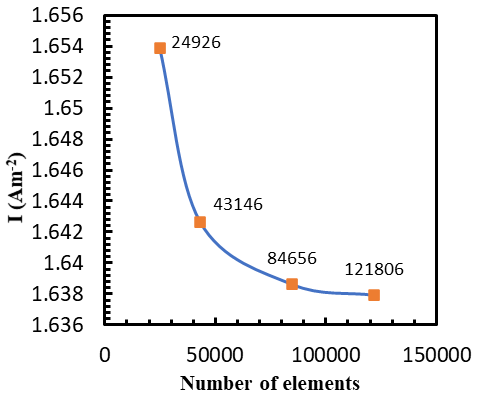


(b)

Fig. 3.(a) Comparison of average current density along the electrodes with different grid sizes, (b) Comparison of multiphysics simulation polarization curves and experimental data.


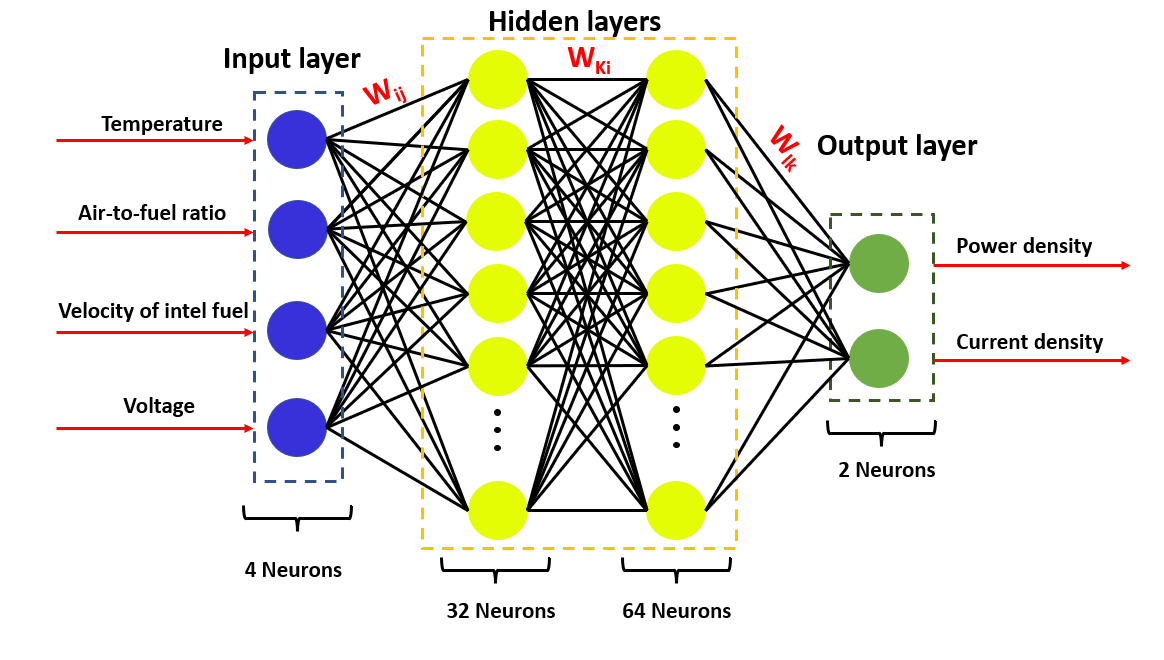


Fig. 4. A structure of ANN with two hidden layers for the current study.

(c)

(a)

(b)


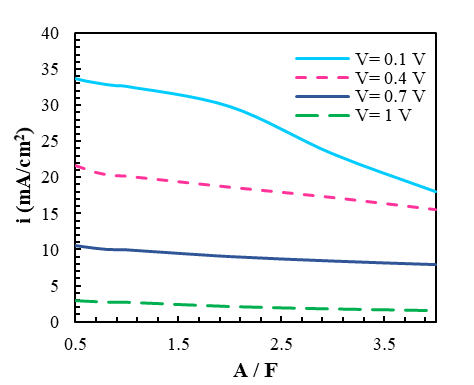

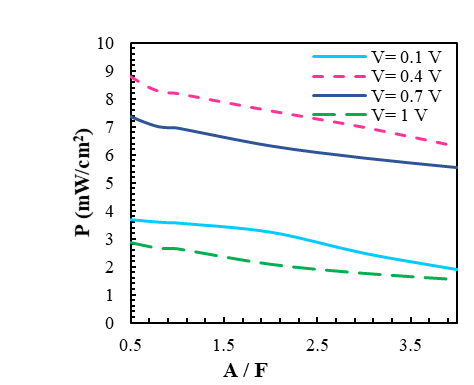


(d)


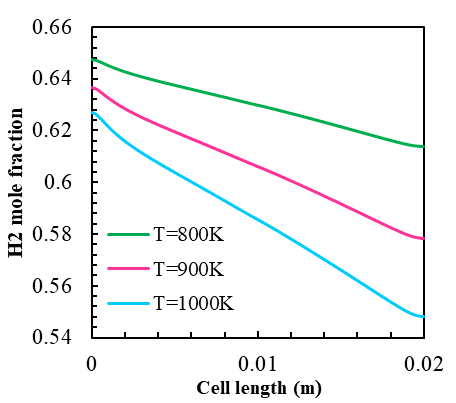

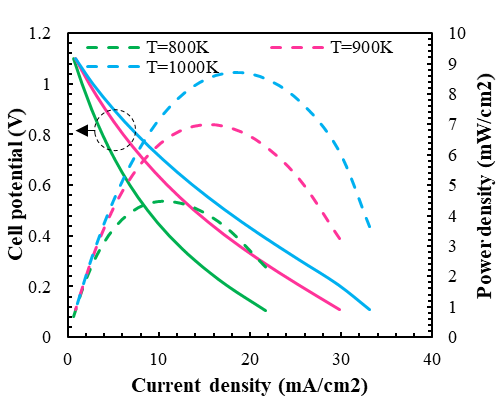


Fig. *5*. (a) The current density distribution at various A/F ratios; (b) distribution of power density at various A/F ratios at a temperature of 973 K*; (c)* V-I and P-I curves with a fuel-to-air ratio of one at different temperatures; (*d*) H2 mole fraction variations at the anode-electrolyte interface for various temperatures.

(c)

(b)

**Flow direction**

**A/F = 0.6**

**A/F = 1**

**A/F = 0.5**


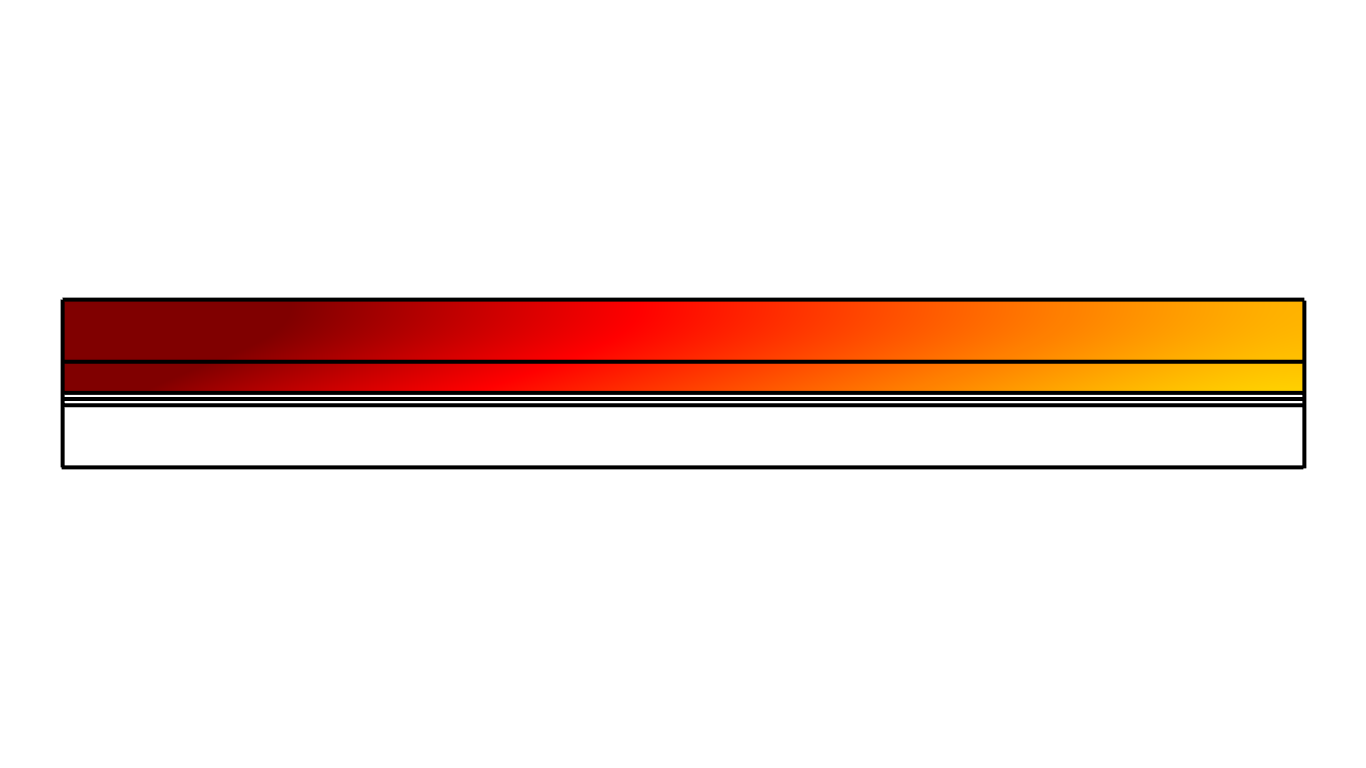

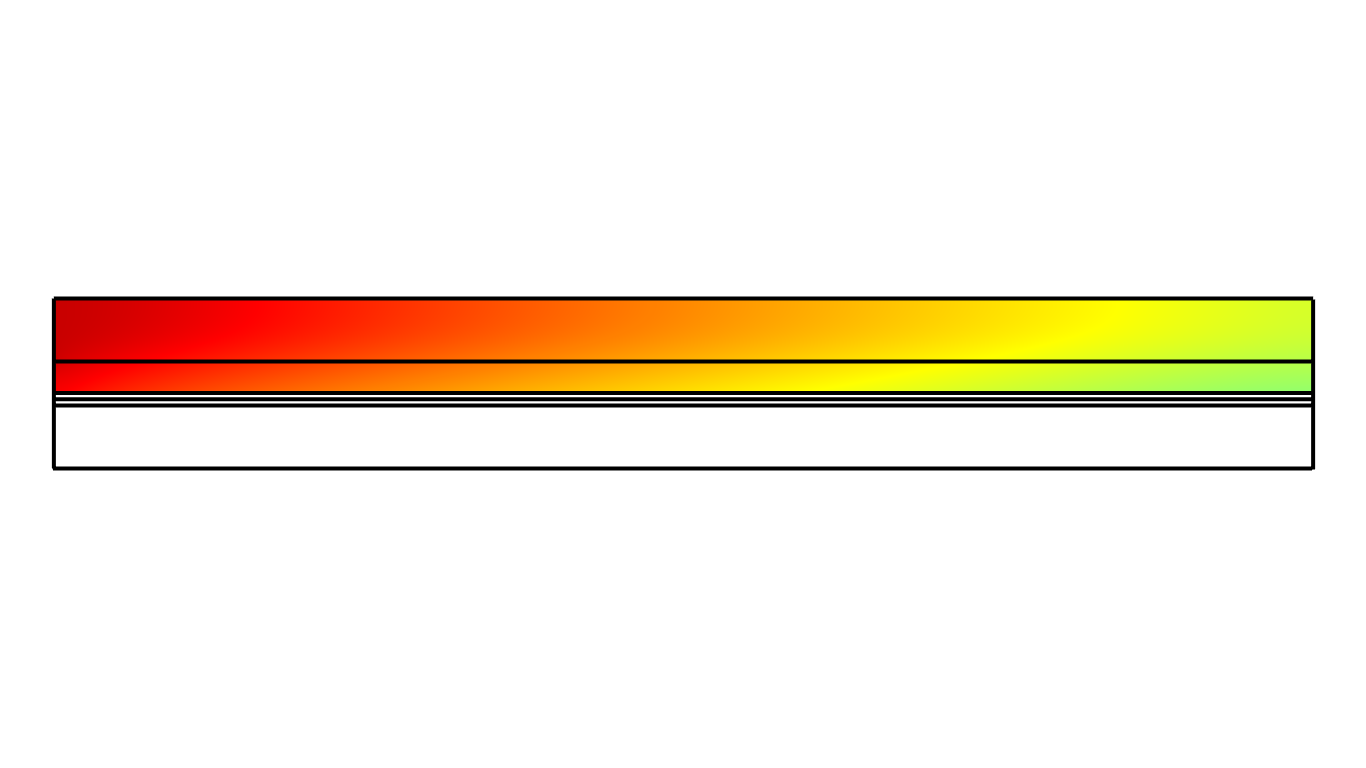

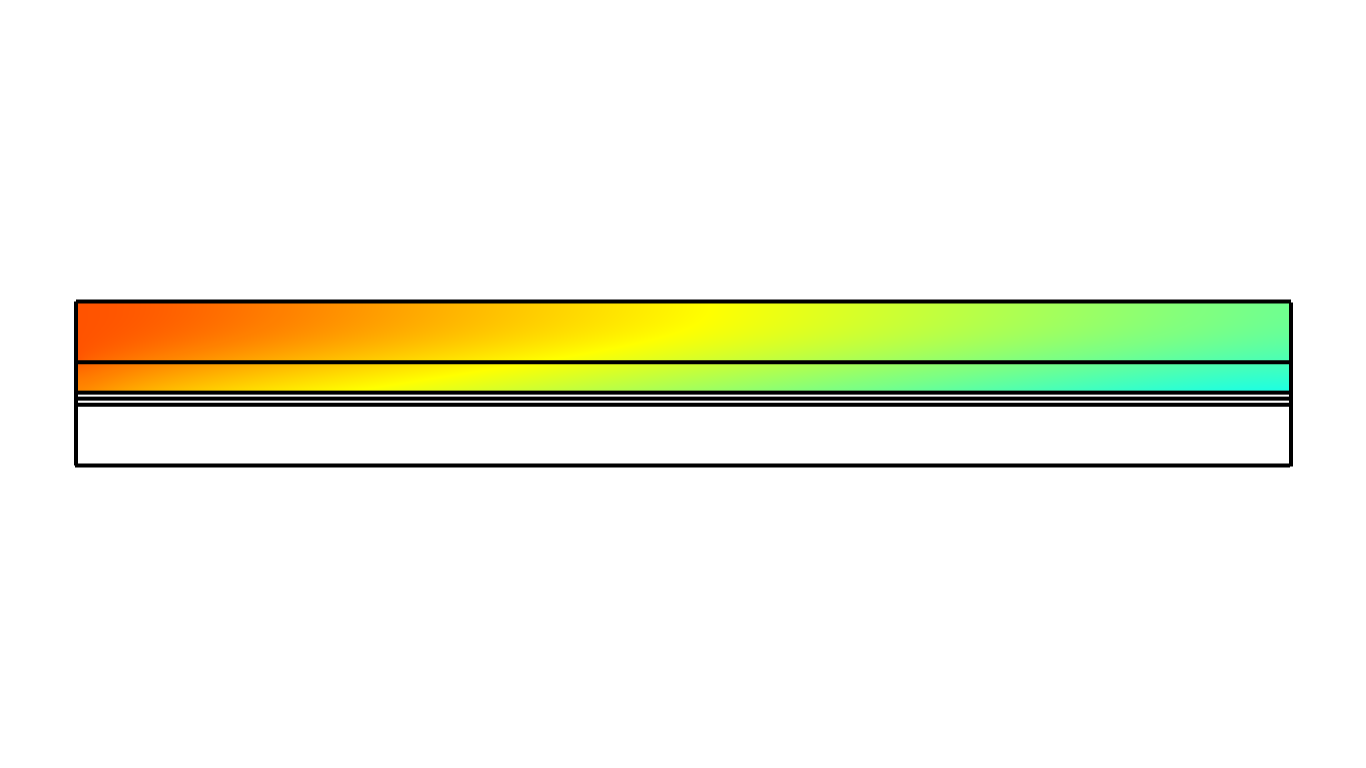

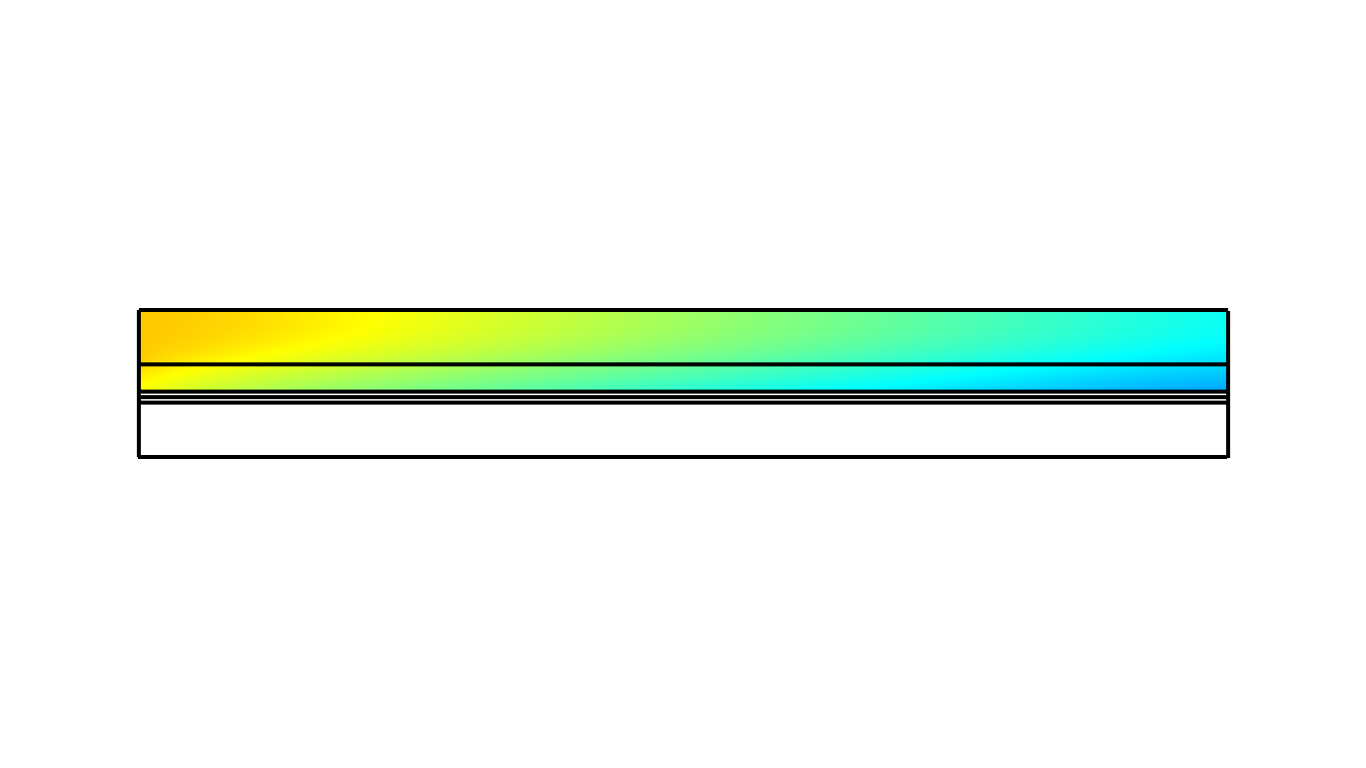

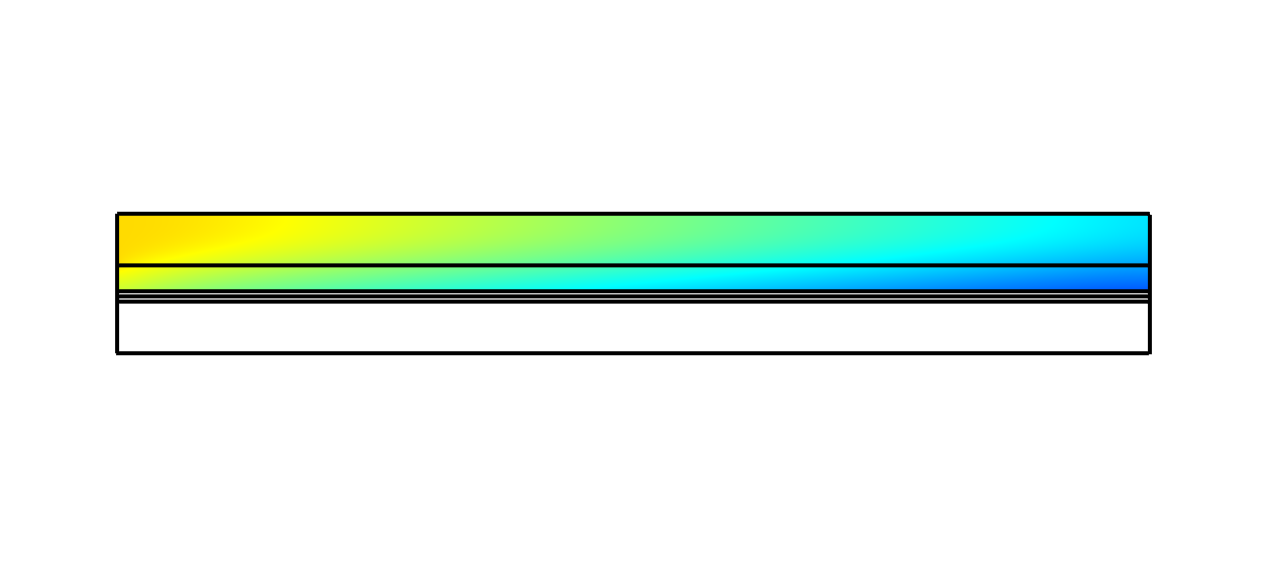

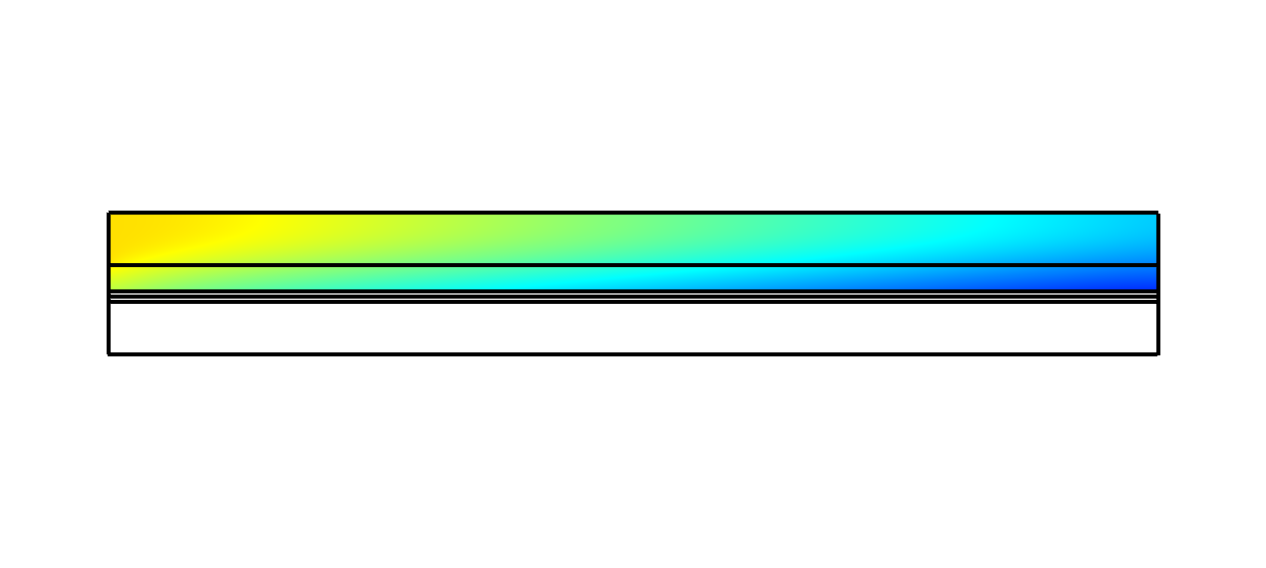


**A/F = 0.7**

**A/F = 0.8**

**A/F = 0.9**


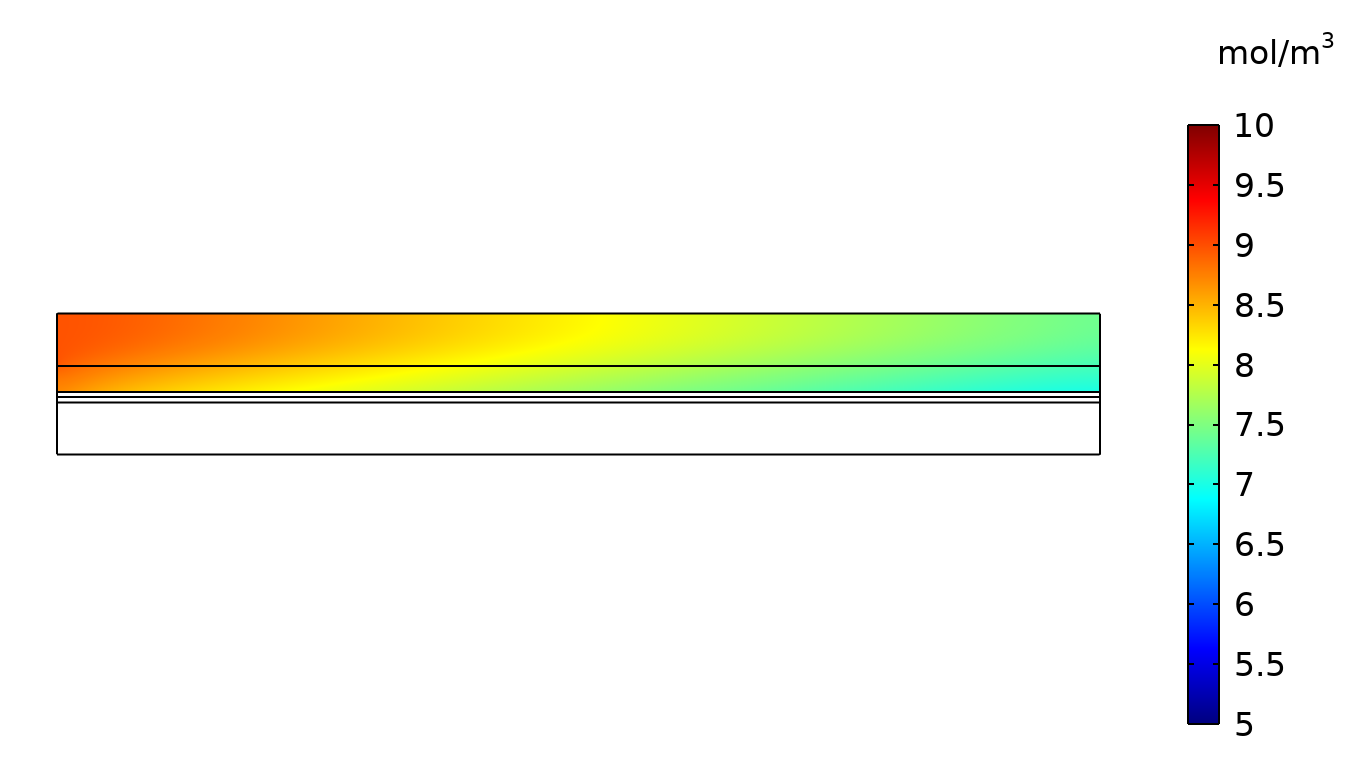

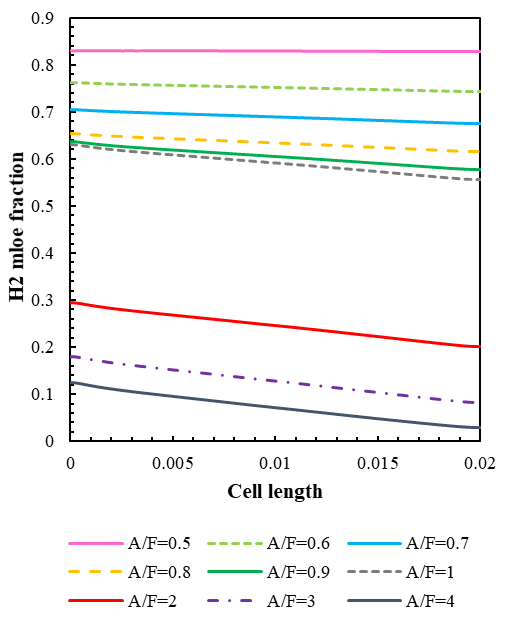


**A/F = 2**

**A/F = 3**

**A/F = 4**


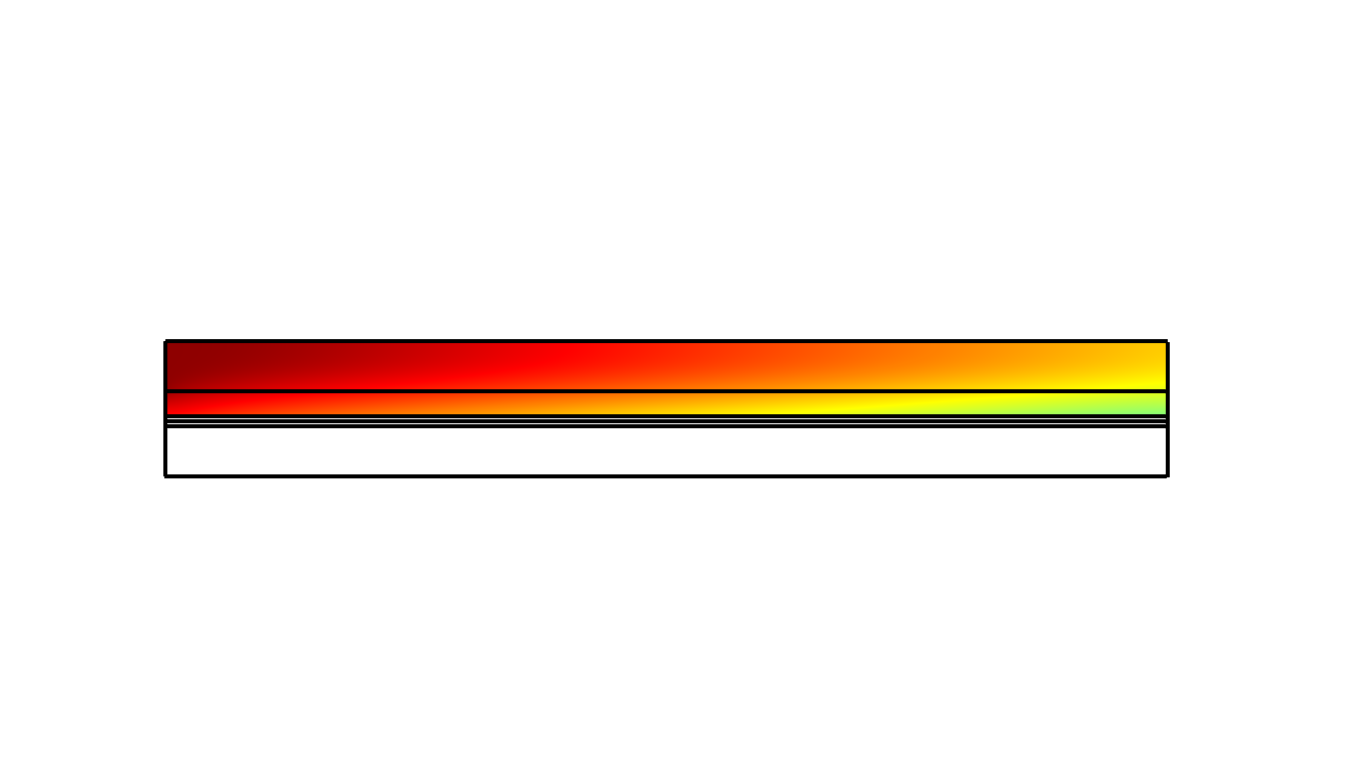

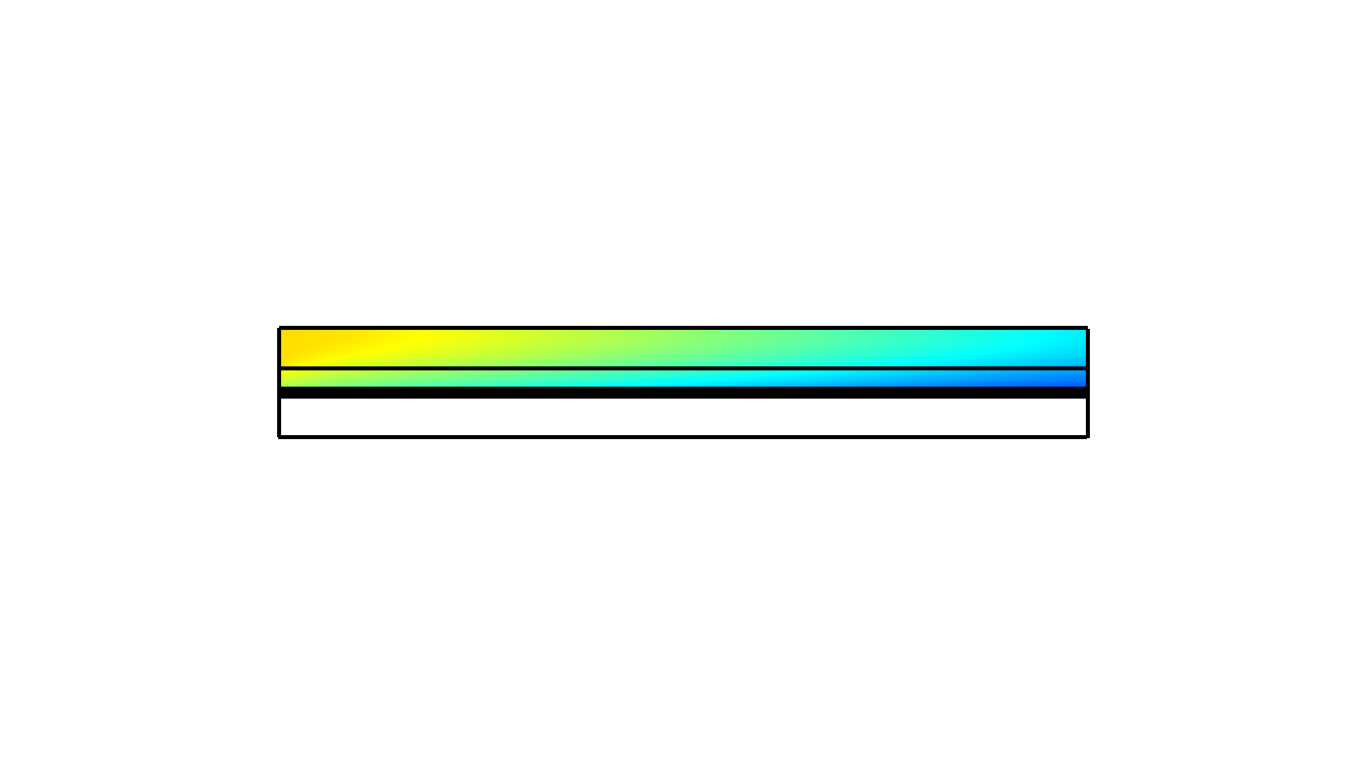

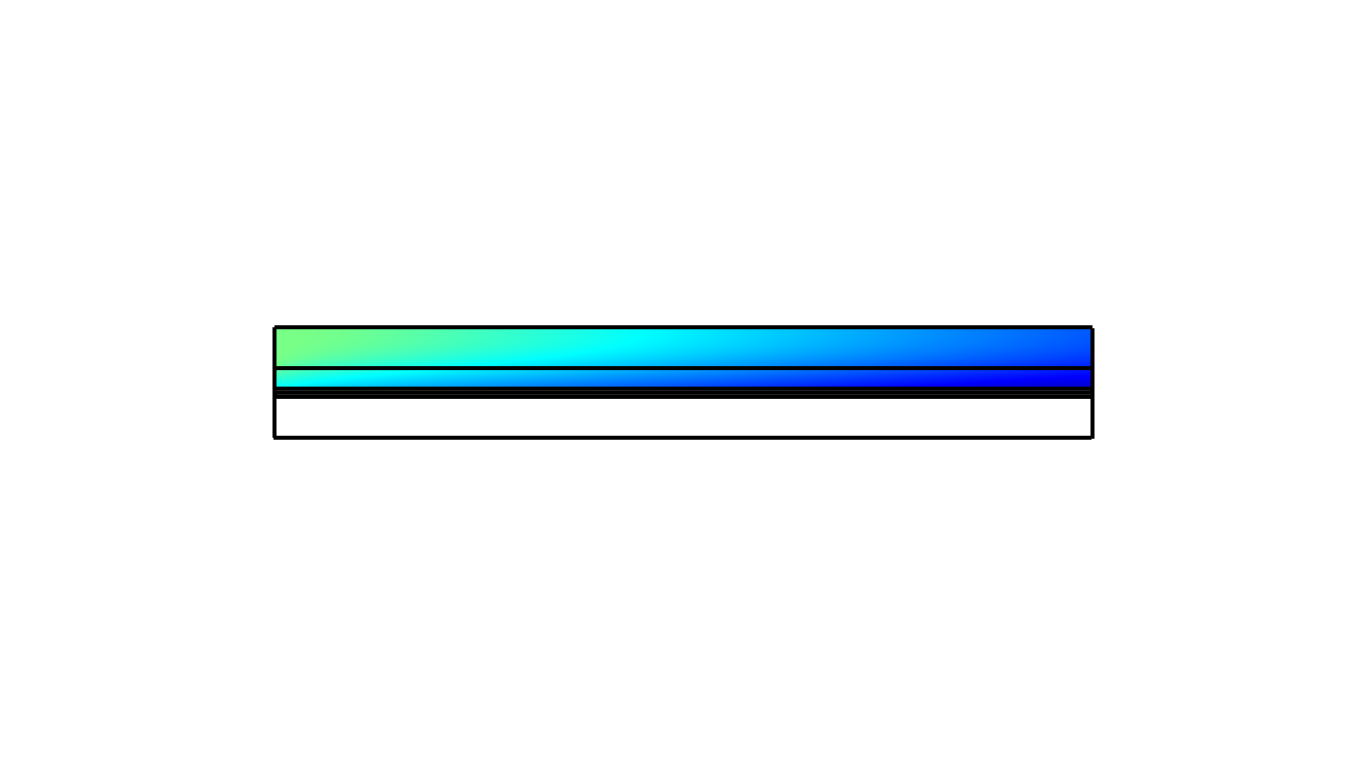

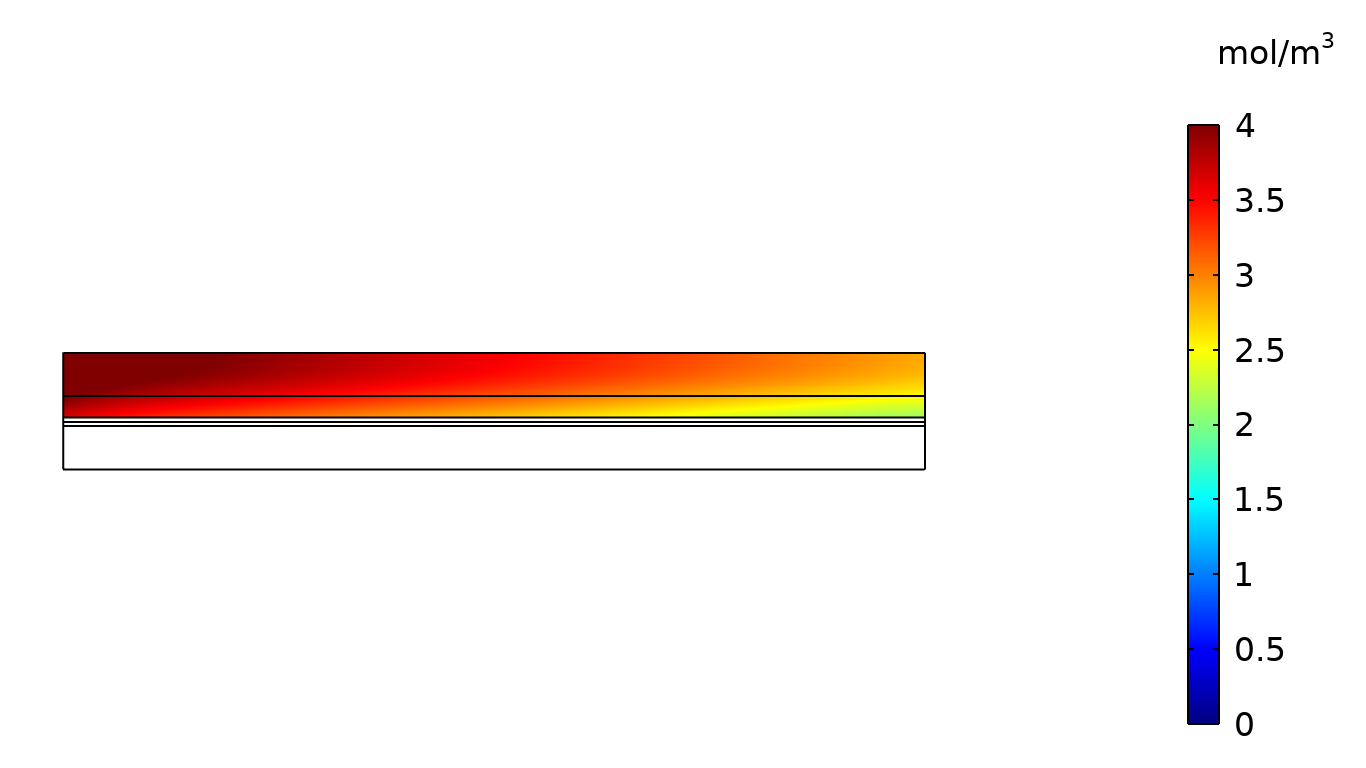


(a)

Fig. 6. (a) H_2_ mole fraction variation at the anode-electrolyte interface with varying A/F ratio at a temperature of 973K, (b); (c) H_2_ mole fraction distribution in the anode and fuel flow channel at a temperature of 973K as a function of different A/F ratios.


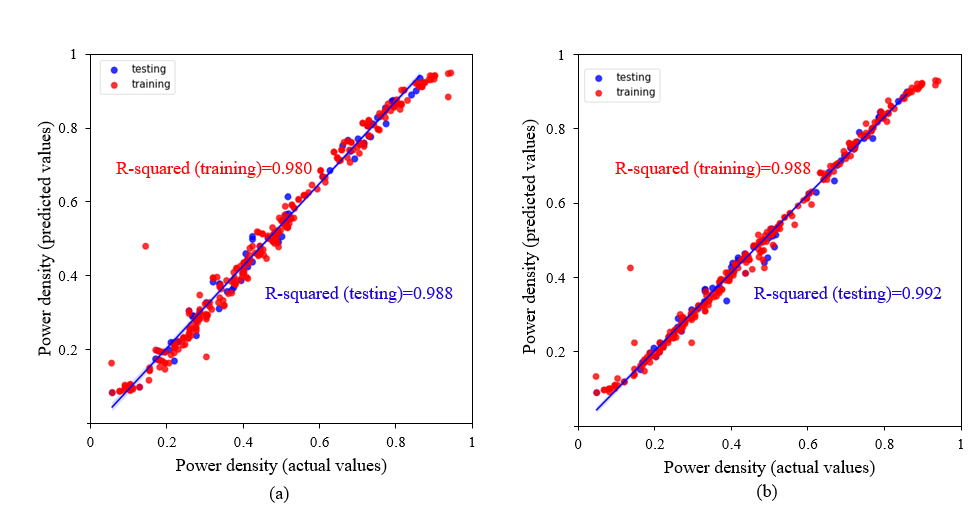


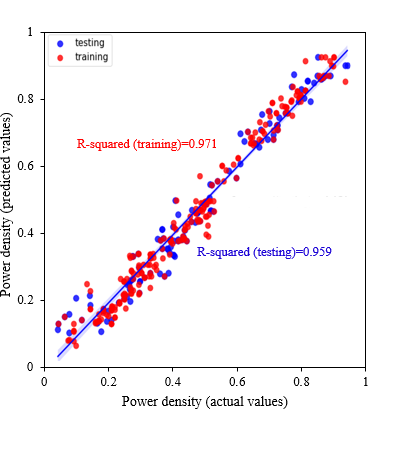


(c)


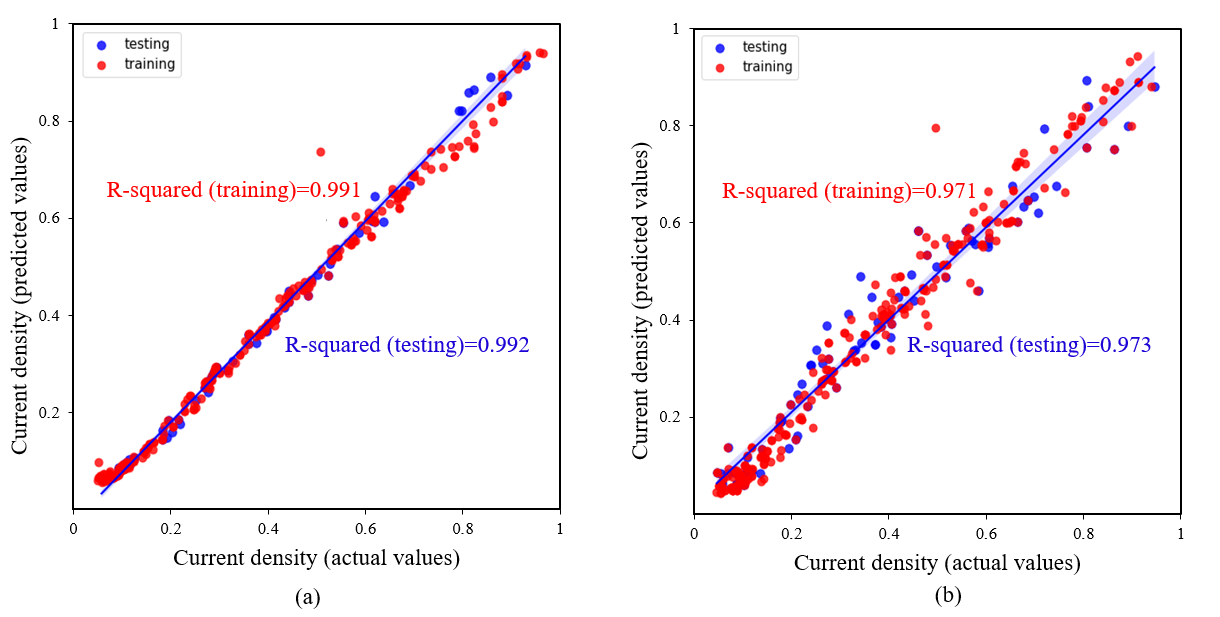


(d)

(e)

Fig. 7. (a) ANN model with three hidden layers and the output parameter P; (b) ANN model with two hidden layers and the output parameter P; (c) The KNN model with K = 3 and the output parameter P; (d) ANN model with two hidden layers and the output parameter I; (e) KNN model with K=3 and the output parameter I.

Fig. 8. The predicted value by the ANN model and its comparison with the actual value obtained from the simulation results (a) of current density and (b) of power density.
